# Singlet oxygen treatment of tumor cells triggers extracellular singlet oxygen generation, catalase inactivation and reactivation of intercellular apoptosis-inducing signaling^☆^

**DOI:** 10.1016/j.redox.2015.07.006

**Published:** 2015-07-17

**Authors:** Michaela Riethmüller, Nils Burger, Georg Bauer

**Affiliations:** Institute of Virology, Department of Medical Microbiology and Hygiene, University Medical Center, Freiburg, Germany

**Keywords:** Singlet oxygen, Photodynamic therapy, Catalase, Peroxnitrite, Nitric oxide, Intercellular apoptosis-inducing signaling

## Abstract

Intracellular singlet oxygen generation in photofrin-loaded cells caused cell death without discrimination between nonmalignant and malignant cells. In contrast, extracellular singlet oxygen generation caused apoptosis induction selectively in tumor cells through singlet oxygen-mediated inactivation of tumor cell protective catalase and subsequent reactivation of intercellular ROS-mediated apoptosis signaling through the HOCl and the NO/peroxynitrite signaling pathway. Singlet oxygen generation by extracellular photofrin alone was, however, not sufficient for optimal direct inactivation of catalase, but needed to trigger the generation of cell-derived extracellular singlet oxygen through the interaction between H_2_O_2_ and peroxynitrite. Thereby, formation of peroxynitrous acid, generation of hydroxyl radicals and formation of perhydroxyl radicals (HO_2_^.^) through hydroxyl radical/H_2_O_2_ interaction seemed to be required as intermediate steps. This amplificatory mechanism led to the formation of singlet oxygen at a sufficiently high concentration for optimal inactivation of membrane-associated catalase. At low initial concentrations of singlet oxygen, an additional amplification step needed to be activated. It depended on singlet oxygen-dependent activation of the FAS receptor and caspase-8, followed by caspase-8-mediated enhancement of NOX activity. The biochemical mechanisms described here might be considered as promising principle for the development of novel approaches in tumor therapy that specifically direct membrane-associated catalase of tumor cells and thus utilize tumor cell-specific apoptosis-inducing ROS signaling.

## Highlights

• Intracellular singlet oxygen causes cell death in nontransformed and in tumor cells.
• Extracellular singlet oxygen causes apoptosis induction selectively in tumor cells.
• Extracellular singlet oxygen triggers the generation of cell-derived singlet oxygen.
• Amplification of singlet oxygen generation causes inactivation of tumor cell catalase.
• Inactivation of catalase reactivates intercellular ROS-dependent apoptosis signaling.

## Graphical abstract

**Figure:**
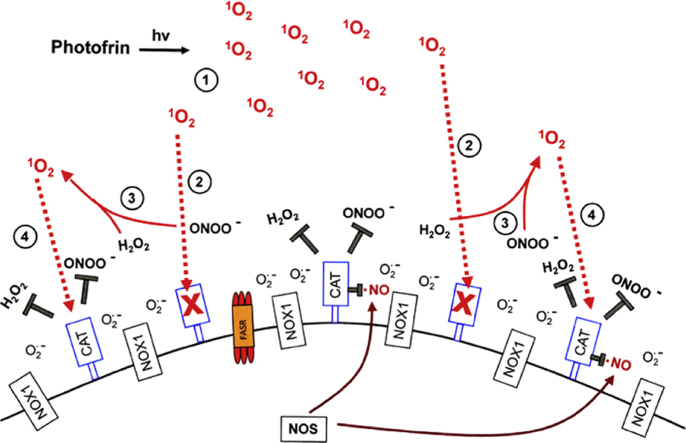

## 1 Introduction

**Fig. 1:**
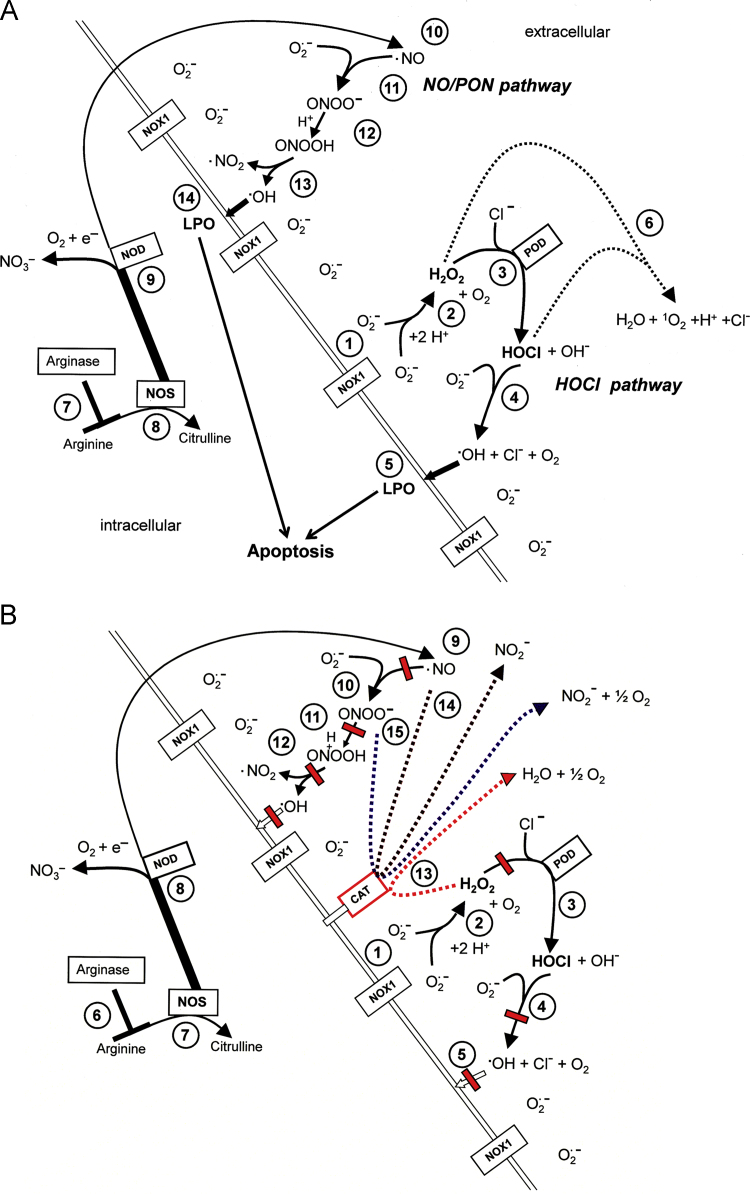
Intercellular apoptosis-inducing ROS signaling. (A) Transformed cells. The figure shows the membrane of a transformed cell with the intracellular space on the left side, the extracellular space on the right side. Transformed cells are defined as malignant cells that have the potential to form tumors but have not yet been confronted with the natural antitumor mechanisms of an organism. Transformed cells are characterized by expression of NOX1 that generates extracellular superoxide anions (#1). These dismutate and form H_2_O_2_ (2O_2_^.–^+2H^+^→H_2_O_2_+O_2_) (#2) which is used by the peroxidase domain of DUOX (POD) as substrate for the generation of HOCl (H_2_O_2_+PODFe^III^→POD Fe^IV^=O^.+^+H_2_O; POD Fe^IV^=O^.+^+Cl^–^+H^+^→PODFe^III^+HOCl) (#3). HOCl interacts with superoxide anions, leading to the generation of hydroxyl radicals (HOCl+O_2_^.–^→.OH+O_2_+Cl^–^) (#4) [24,31,32] that induce lipid peroxidation (#5) and subsequent apoptosis induction through the mitochondrial pathway of apoptosis. In the presence of an high excess of H_2_O_2_ compared to POD, a consumption reaction between H_2_O_2_ and HOCl (#6) blunts HOCl signaling. The level of arginine is controlled by arginase (#7). NO synthase (NOS) utilizes arginine as substrate for the synthesis of NO (#8) [33-35]. A substantial part of NO may be converted into nitrate by NO dioxygenase (#9), which is connected to the activity of cytochrome P 450 oxidoreductase (POR). NO passes the cell membrane (#10) and reacts with superoxide anions, resulting in the formation of peroxynitrite (.NO+O_2_^**.–**^→ONOO^–^) (#11) [36–40]. Protonation of peroxynitrite leads to the formation of peroxynitrous acid (ONOO^–^+H^+^→ONOOH→.NO_2_+.OH) (#12) [37,41–43]. As malignant cells have efficient proton pumps that establish a high local concentration of protons on the outside of their cell membrane [44], the formation of ONOOH seems to be locally favored over the competing reaction between ONOO^−^ and CO_2_ (ONOO^−^ and CO_2_→ONOOCOO^−^→NO_2_+CO_3_^−^) [45–48]. Peroxynitrous acid spontaneously decomposes into NO_2_ and hydroxyl radicals (#13), which induce lipid peroxidation and the mitochondrial pathway of apoptosis (# 14). (B) Tumor cells (defined as malignant cells derived from a *bona fide* tumor) are protected against intercellular apoptosis-inducing ROS signaling through expression of membrane-associated catalase. Tumor progression causes the selection of a phenotype that is characterized by the expression of membrane-associated catalase [54,56]. Membrane-associated catalase protects the tumor cells against ROS signaling by the HOCl pathway (#1–#5) and the NO/peroxynitrite pathway (#6–#12) through decomposition of H_2_O_2_ (#13), oxidation of NO (#14) and decomposition of peroxynitrite (#15). Decomposition of H_2_O_2_ and peroxynitrite by catalase are two step reactions with compound I (CATFe^IV^=O^**.+**^) as intermediate. NO is oxidated to NO_2_^**−**^ by compound I.

Reactive oxygen and nitrogen species (ROS) can cause destructive effects through their mutagenic potential and through their ability to react with proteins and lipids, but they can also establish specific and fine-tuned signaling pathways [1,2]. During multistep oncogenesis, reactive oxygen and nitrogen species establish remarkable signaling pathways that induce oncogenic- and antioncogenic effects. Extracellular superoxide anions generated by membrane-associated NADPH oxidase (NOX) and their dismutation product H_2_O_2_ control the proliferation of malignant cells and are involved in the maintenance of the transformed state in vitro [3–8] and in vivo [8–14]. These oncogenic effects of superoxide anions are counteracted by ROS-dependent intercellular induction of apoptosis, a process that selectively eliminates transformed cells [1,15–30]. ROS-dependent intercellular apoptosis induction is mainly based on the HOCl [1,23,24,29,30] and the NO/peroxynitrite signaling pathway [1,24,26,29,30]. In both pathways, extracellular superoxide anions derived from NOX1 determine the efficiency and the selectivity of apoptosis-inducing signaling. Please find details in Fig. 1A. Tumor progression in vivo requires the acquisition of the “H_2_O_2_-catabolizing phenotype”, i.e. resistance against ROS-mediated apoptosis induction [49–53]. This resistance is established through expression of membrane-associated catalase, which interferes with HOCl signaling through decomposition of H_2_O_2_ [54–56], and with NO/peroxynitrite signaling through oxidation of NO [57] and decomposition of peroxynitrite [56,58] (Fig. 1B). The combination of the two phenotypic features “NOX-dependent generation of extracellular superoxide anions” and “protection by membrane-associated catalase” has been found to be a regular trait in all human and rodent tumor cell lines tested [30]. This trait has been suggested to be potentially useful for novel approaches for antitumor therapy that are based on inhibition or inactivation of catalase and restoration of specific apoptosis-inducing ROS signaling of the malignant cells [29,30].

Classical photodynamic therapy of tumors is based on the *intracellular* localization of photosensitizers preferentially in tumor tissue. Upon photoactivation, the photosensitizers generate singlet oxygen (^1^O_2_) which induces apoptosis or necrosis [59]. Due to the high reactivity of singlet oxygen, a multitude of intracellular targets may be hit. Finally, this may lead to the induction of the mitochondrial pathway of apoptosis. It has also been shown that singlet oxygen can inactivate antioxidant enzymes like catalase or SOD through interaction with critical histidine residues in their active centers [60,61]. However selective photodynamic therapy based on induction of oxidative stress through inactivation of antioxidant enzymes that specifically protect tumor cells from intercellular ROS-mediated apoptosis signaling has not yet been established or suggested to our knowledge.

Recent results from our group have shown that extracellular singlet oxygen generated through the interaction between cell-derived H_2_O_2_ and peroxynitrite [62] has the potential to inactivate membrane-associated catalase that protects tumor cells from intercellular ROS signaling [29,63,64] and thus reactivates ROS-dependent apoptosis induction selectively in tumor cells. The details of the reactions between H_2_O_2_ and peroxynitrite that lead to the generation of singlet oxygen [62,65,66] will be further discussed under Supplementary materials [Supplementary Figs. 4–6].

Formation of cell-derived singlet oxygen required an initial local inactivation of a few catalase molecules on the surface of tumor cells. This was triggered through an increase in free NO. NO-dependent partial and reversible inhibition of catalase then seemed to allow the first round of singlet oxygen formation through H_2_O_2_/peroxynitrite interaction, as it prevented the decomposition of these two catalase substrates. Even if the concentration of singlet oxygen reached was suboptimal for substantial inactivation of a sufficient subpopulation of protective catalase molecules, it seemed to be sufficient to activate the FAS receptor in a ligand-independent mode, according to the findings described by Zhuang et al. [67]. As a result, caspase-8 was activated and, according to published work [68–70] enhanced NOX activity and possibly also NO synthase (NOS) induction. The resultant increased generation of superoxide anions, H_2_O_2_, NO and peroxynitrite then seemed to be sufficient to generate an optimal concentration of singlet oxygen that was required for catalase inactivation. When NOX was stimulated by treatment of the cells with TGF-beta or low dose radiation [71], the requirement for caspase-8 activity became dispensable [63,64]. Due to the relatively low concentration of the FAS receptor in the cell systems studied, direct activation of the FAS receptor-dependent cell death pathway did not substantially contribute to overall apoptosis induction.

In the study presented here, direct application of photofrin-derived singlet oxygen was used as experimental approach. It was performed in order to verify or falsify the proposed specific antitumor action of singlet oxygen through catalase inactivation and reactivation of intercellular ROS signaling that had been derived by previous cell biological experiments [63,64]. Our study was also performed to clarify whether targeting of *extracellular* catalase of tumor cells by photodynamic therapy might be useful to improve rational and selective tumor therapy.

## 2 Materials and methods

### 2.1 Materials

4-(2-Aminoethyl)benzenesulfonyl fluoride (AEBSF), 3-aminotriazole (3-AT), mannitol, neutralizing monoclonal antibodies against catalase (clone CAT-505, mouse, IgG1), monoclonal antibodies directed against laminin, monoclonal antibodies (clone DX2) directed against human FAS receptor (Apo-1/CD95), purified catalase from bovine liver, the NOS inhibitor N-omega-nitro-l-arginine methylester hydrochloride (l-NAME), taurine, Mn-SOD from E. coli, Cu/Zn-SOD from bovine erythrocytes, and histidine were obtained from Sigma-Aldrich (Schnelldorf, Germany). The peroxidase inhibitor 4-aminobenzoyl hydrazide (ABH) was obtained from Acros Organics (Geel, Belgium). The catalase mimetic EUK 134 [chloro([2,2′-[1,2-ethanediylbis[(nitrilo-κN)methylidyne]]bis[6-methoxyphenolato-κO]]]-manganese) was a product of Cayman and was obtained from Biomol (Hamburg, Germany). Inhibitors for caspase-3 (Z-DEVD-FMK), caspase-8 (Z-IETD-FMK) and caspase-9 (Z-LEHD-FMK) were obtained from R&D Systems (Wiesbaden-Nordenstadt, Germany).

Peroxynitrite and the peroxynitrite decomposition catalyst 5-, 10-, 15-, 20-Tetrakis(4-sulfonatophenyl)porphyrinato iron(III) chloride (FeTPPS) were obtained from Calbiochem (Merck Biosciences GmbH, Schwalbach/Ts, Germany). Photofrin (a product of Axcan, Canada) was obtained from Meduna Arzneimittel GmbH (Aschaffenburg, Germany). TGF-beta1 was purified from human platelets as recently described [15].

Detailed information on inhibitors has been previously published [26,54,64,72,75]. The site of action of inhibitors and scavengers is shown in the supplementary material of Bauer et al. [75] and Bauer and Zarkovic [64].

Mn-SOD was used as superoxide anion scavenger in all inhibition experiments, as this enzyme is characterized by strong inhibitory potential over a wide concentration range of superoxide anions [75]. In contrast, Cu/Zn-SOD is characterized by a bell-shaped inhibition curve that is not adequate for inhibition experiments but can be instrumentalized for the quantitation of the relative concentration of superoxide anions (see below).

Preparation of photofrin dilutions and their addition to the assays was performed at dimmed out light. Singlet oxygen generation by photofrin was induced by illumination with visible light under the working bench for the indicated times.

### 2.2 Cells and media for cell culture

The human gastric adenocarcinoma cell line MKN-45 (ACC 409) (established from the poorly differentiated adenocarcinoma of the stomach (medullary type) of a 62 year-old woman) was purchased from DSMZ, Braunschweig, Germany. The human Burkitt lymphoma cell line GUMBUS (established from the liquor of a 28-year-old man with European Burkitt lymphoma at second relapse that became highly resistant to several chemotherapy regimens) (ACC630) was obtained from Dr. Doelken, University of Greifswald, Germany. The murine fibrosarcoma cell line L929 was obtained from Dr. D. Adam, Kiel, Germany. Nontransformed diploid fibroblasts Alpha-1 have been established in our institute from the foreskin of a healthy proband. The cells enter senescence after 30–40 passages and have been shown to be free of NOX1 expression and extracellular catalase [56]. MKN-45 and GUMBUS were cultured in RPMI 1640 medium, containing 10% fetal bovine serum (FBS) plus supplements. L929 and Alpha-1 cells were cultivated as adherent cultures in Eagle's Minimum Essential Medium (EMEM), supplemented with 5% heat-treated FBS and supplements, as described in references [56,75].

## 3 Methods

### 3.1 Apoptosis induction

#### 3.1.1 Autocrine apoptosis induction by intercellular ROS signaling (Figs. 2, 3, 7)

**Fig. 2:**
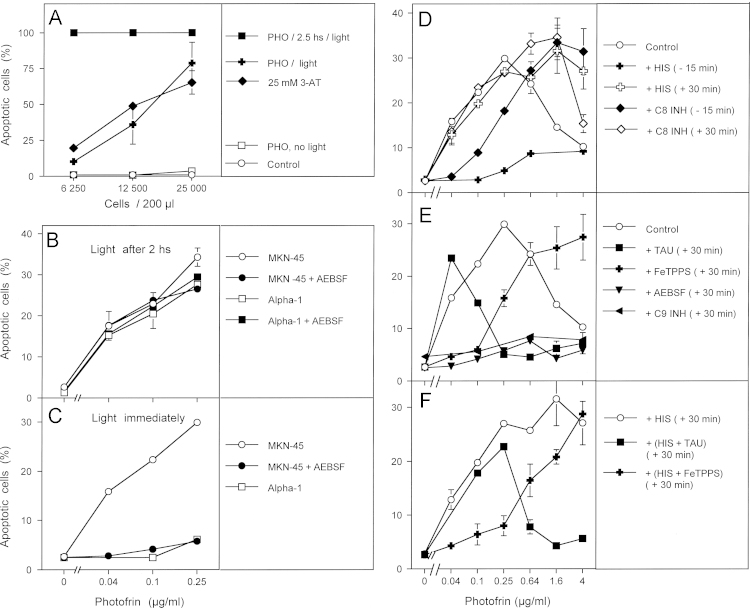
Differential effects of intra- and extracellularly generated singlet oxygen and the differentiation between early and late effects during photofrin-mediated apoptosis induction. (A) The indicated concentrations of the murine fibrosarcoma cell line L929 received either 25 mM 3-AT, 10 μg/ml photofrin or remained without additions (control). Photofrin-containing assays were either not illuminated or illuminated with visible light immediately after addition of photofrin or 2.5 h after addition of photofrin. The percentages of apoptotic cells were determined after 48 h. (B, C) 12,500 MKN-45 gastric carcinoma cells or 10,000 Alpha-1 normal human diploid fibroblasts per assay, in the absence or presence of 100 μM AEBSF were either illuminated 2 h after addition of the indicated concentrations of photofrin (B) or immediately after addition of photofrin (C). The percentages of apoptotic cells were determined after 3.5 h. (D–F) 12,500 MKN-45 cells per assay were either free of inhibitors (control) or received the indicated inhibitors either 15 min before or 30 min after addition of the indicated concentrations of photofrin. Illumination with visible light was for 30 min. The percentages of apoptotic cells were determined 3.5 h after illumination. HIS: 2 mM histidine; C8 INH: 25 μM caspase-8 inhibitor; TAU: 50 mM taurine; FeTPPS: 25 µM; AEBSF: 100 µM; C9 INH: 25 mM caspase9 inhibitor. Statistical analysis: (A) Apoptosis induction by photofrin and 3-AT were highly significant (*p*<0.001). There was no statistically significant difference between the effect of 3-AT and photofrin. (B) No significant differences. (C) Apoptosis induction in MKN-45 and its inhibition by AEBSF were highly significant (*p*<0.001), whereas there was no significant apoptosis induction in non-malignant Alpha-1 cells. (D–F) Apoptosis induction by photofrin was highly significant (*p*<0.001). All inhibitory effects mentioned in the text were highly significant (*p*<0.001).

Cells in complete medium were seeded in 96-well tissue culture clusters at a density of 12,500 cells/100 µl (MKN-45), 25,000 cells/100 µl (GUMBUS) or 10,000 cells/100 µl (Alpha-1). L929 cells were seeded at the indicated concentrations in 200 µl complete medium in 48-well tissue culture clusters (Fig. 2A). Tumor cell catalase was either inhibited by the indicated concentration of 3-aminotriazole (3-AT) or by singlet oxygen-mediated inactivation after treatment with the indicated concentrations of photofrin and illumination with visible light for 20 min at room temperature. In all experiments, assays were performed in duplicate. After the indicated time of incubation at 37 °C and 5% CO_2_ that allowed intercellular ROS-mediated apoptosis induction, the percentage of apoptotic cells was determined by inverted phase contrast microscopy based on the classical criteria for apoptosis, i.e., nuclear condensation/fragmentation or membrane blebbing [56,73,74]. The characteristic morphological features of intact and apoptotic cells, as determined by inverted phase contrast microscopy have been published [22,56,75]. At least 200 neighboring cells from randomly selected areas were scored for the percentage of apoptotic cells at each point of measurement. Control assays ensured that the morphological features “nuclear condensation/fragmentation” as determined by inverse phase contrast microscopy were correlated to intense staining with bisbenzimide and to DNA strand breaks, detectable by the TUNEL reaction [15,22,26].

#### 3.1.2 Apoptosis induction mediated by exogenous peroxynitrite (Figs. 4–6)

Treatment with exogenous peroxynitrite allows to quantitatively monitor the activity of membrane-associated catalase as this enzyme decomposes exogenous peroxynitrite, whereas intracellular catalase cannot reach exogenous peroxynitrite before the compound attacks the cell membrane [56]. After the indicated pretreatments at a density of 125,000 cells/ml, the cells were washed several times through centrifugation and resuspension in fresh medium and then were either seeded at a density of 4000 cells/100 µl (Figs. 4 and 5) or 12,500 cells/100 µl (Fig. 6). Cells seeded at 12,500 cells/100 µl received 100 µM AEBSF to prevent autocrine apoptosis induction, cells seeded at 4000 cells/100 µl were kept without AEBSF as their low density prevented autocrine apoptosis induction. Peroxynitrite was diluted in ice-cold PBS immediately after controlled thawing and was rapidly applied to the cells. This approach allows to focus on apoptosis induction by exogenous peroxynitrite which is an indication for the inactivation of membrane-associated catalase. Apoptosis criteria were used as defined above.

#### 3.1.3 Quantitation of superoxide anion concentration (Fig. 7)

The quantification of superoxide anion concentrations was based on the superoxide anion-dependent induction of apoptosis and its inhibition by Cu/Zn-SOD as recently described [71]. In the experiment presented in Fig. 7, apoptosis was induced in MKN-45 cells through the catalase inhibitor 3-AT, photofrin-treatment or monoclonal antibodies directed against the FAS receptor, as described in detail in the legend. As verified in control experiments, apoptosis induction by these treatments was dependent on superoxide anions and therefore can be inhibited by Cu/Zn-SOD. The inhibition of superoxide anion-dependent apoptosis induction by Cu/Zn-SOD resulted in a bell-shaped curve [71,75]. This specific feature of the action of Cu/Zn-SOD facilitates the relative quantification of the extracellular superoxide anion concentration. Control experiments confirmed that an increase in superoxide anion concentration also caused a linear increase in the concentration of Cu/Zn-Sod necessary for optimal inhibition which thus can be taken as an arbitrary equivalent of the superoxide anion concentration.

### 3.2 Statistics

#### 3.2.1 . Statistical analysis

In all experiments, assays were performed in duplicate and empirical standard deviations were calculated. Absence of standard deviation bars indicates that the standard deviation was too small to be reported by the graphic program. Empirical standard deviations merely demonstrate reproducibility in parallel assays but do not allow statistical analysis of variance. The experiments have been repeated at least twice (with duplicate assays). The Yates continuity corrected chi-square test was used for the statistical determination of significances (*p*<0.01=significant; *p*<0.001=highly significant).

## 4 Results

Illumination of murine fibrosarcoma cells that had been loaded with the photosensitizer photofrin for 2.5 h caused massive cell death independent of the original density of the cells. In contrast, illumination immediately after addition of photofrin, i.e. before it had a chance to enter the cells, caused apoptosis-induction dependent on the cell density. In this respect, it was analogous to the action of the catalase inhibitor 3-aminotriazole (3-AT) (Fig. 2A). Addition of photofrin without illumination had no apoptosis-inducing effect, confirming the role of singlet oxygen for the biological effect observed. These findings indicate that photofrin-dependent singlet oxygen generation within the cells caused apoptosis induction directly, whereas singlet oxygen generated outside the cells seemed to trigger an apoptosis-inducing intercellular signaling mechanism, as indicated by its dependence on the density of the cells. Treatment of human gastric carcinoma cells MKN-45 and human normal diploid fibroblasts Alpha-1 with increasing concentrations of photofrin, followed by illumination two hours after addition of photofrin, caused apoptosis induction dependent of the concentration of photofrin and with similar efficiency in malignant and nonmalignant cells (Fig. 2B). Apoptosis induction under these conditions was not inhibited by the NOX1 inhibitor AEBSF, indicating that NOX1-derived extracellular superoxide anions played no role for apoptosis induction under these conditions. When tumor cells and normal cells were illuminated immediately after addition of photofrin (Fig. 2C), apoptosis was induced selectively in the tumor cells in a superoxide anion-dependent process, as it was inhibited by the NOX inhibitor AEBSF. In contrast, nonmalignant diploid fibroblasts that lack sustained NOX1 activity were not affected.

If this assumption was true, the effect of exogenous singlet oxygen should first cause inactivation of membrane-associated catalase, followed by reactivated intercellular ROS signaling. We therefore aimed to analyze these two processes separately by differential addition of specific inhibitors and scavengers in relation to the time point of photofrin addition. Addition of increasing concentrations of photofrin to MKN-45 human gastric carcinoma cells, followed by immediate illumination, caused concentration-dependent apoptosis induction in the mode of an optimum curve (Fig. 2C and D). The differential addition of histidine and caspase-8 inhibitor with respect to time defined an early step during the first 30 min, that was (i) dependent on singlet oxygen over the whole concentration range of photofrin, and (ii) dependent on caspase-8 in the low concentration range of photofrin. In contrast, the NOX inhibitor AEBSF, the HOCl scavenger taurine, the peroxynitrite decomposition catalyst FeTPPS, as well as caspase-9 inhibitor still caused inhibition when they were applied 30 min after addition of photofrin, i.e. immediately after the end of the illumination. The inhibitor profile indicated that low concentrations of photofrin allowed NO/peroxynitrite signaling (inhibited by AEBSF, caspase-9 inhibitor, FeTPPS, but not taurine), whereas higher concentrations allowed HOCl signaling (inhibited by AEBSF, caspase-9 inhibitor and taurine, but not by FeTPPS). When histidine had been added 30 min after photofrin, the optimum curve of apoptosis induction shifted to a plateau-type curve, with a longer concentration range characterized by NO/peroxynitrite signaling and a shorter concentration range with HOCl signaling, compared to control.

**Fig. 3:**
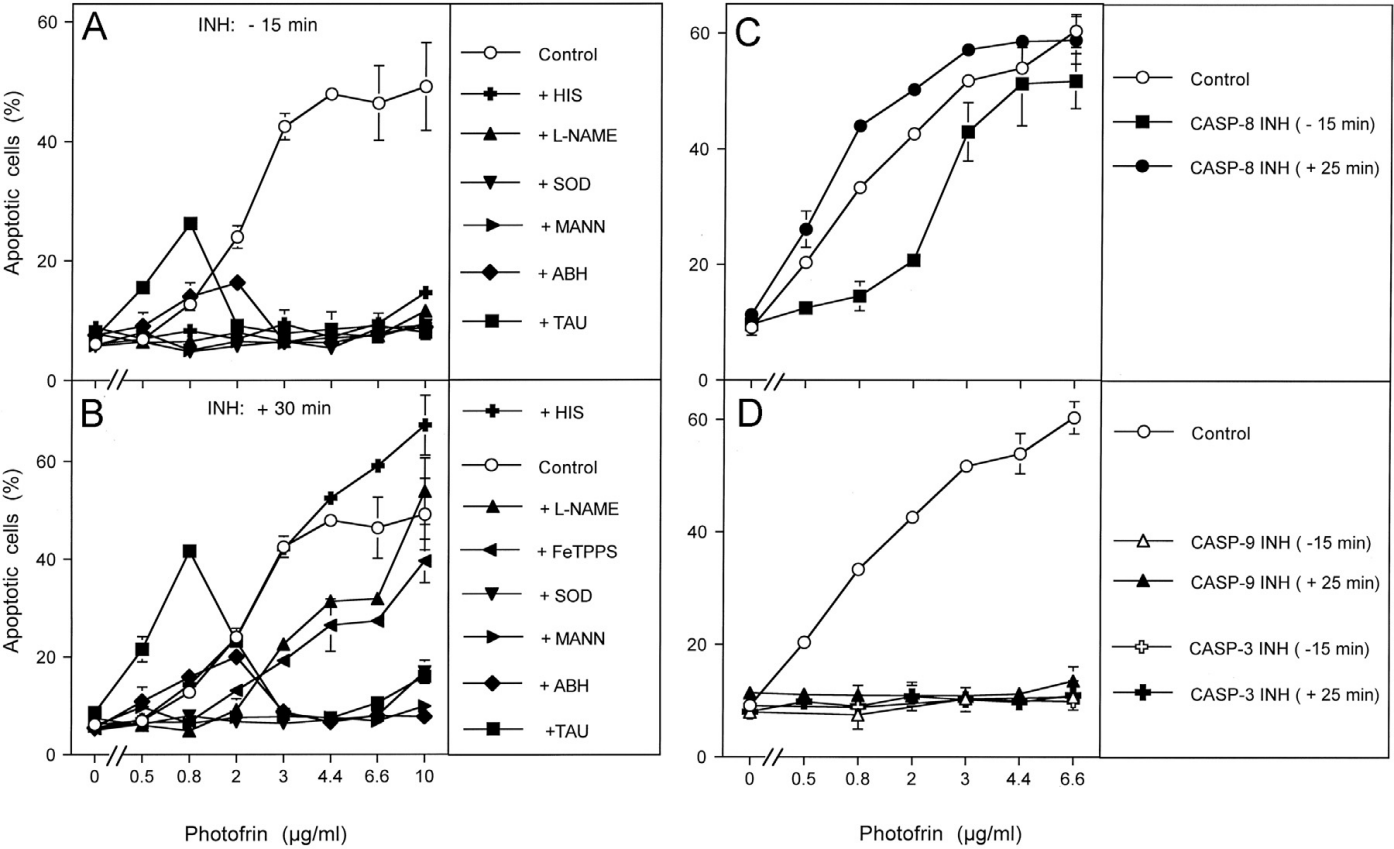
Differentiation between early and late effects during photofrin-mediated apoptosis induction in the human lymphoma cell line GUMBUS. (A, B) 25,000 GUMBUS cells per assay were either free of inhibitors (control) or received the indicated inhibitors either 15 min before or 30 min after addition of the indicated concentrations of photofrin. Illumination with visible light was for 30 min. The percentages of apoptotic cells were determined 2.5 h after illumination. HIS: 2 mM histidine; l-NAME: 2.4 mM; SOD: 100 U/ml; MANN: 10 mM mannitol; ABH: 150 μM; TAU: 50 mM taurine. (C, D) 25,000 GUMBUS cells per assay remained free of inhibitors (control) or received 25 μM caspase 8 inhibitor, 25 mM caspase-9 inhibitor or 50 μM caspase-3 inhibitor either 15 min before or 25 min after addition of photofrin. The assays were illuminated with visible light for 30 min and the percentages of apoptotic cells were determined after additional 2.5 h. Statistical analysis: (A, B) Apoptosis induction was highly significant (*p*<0.001). Inhibition by all inhibitors was highly significant in the concentration ranges mentioned in the text. The differences between early (−15 min) and late (+30 min) addition of histidine, l-NAME and FeTPPS were highly significant (*p*<0.001), whereas all other inhibitors were acting independent of the time of addition. (C) Apoptosis induction by photofrin (0.8–6.6 μg/ml) and its inhibition by caspase-8 inhibitor at 0.8 and 2 μg/ml were highly significant (*p*<0.001). (D) Apoptosis induction by photofrin (0.8–6.6 μg/ml) and its inhibition were highly significant (*p*<0.001).

As NOX1 expression and the expression of membrane-associated catalase has been found to be a regular feature of tumor cells [30], photofrin-derived singlet oxygen should induce the same sequence of biochemical steps in tumor cells other than MKN-45 as well. Treatment of the human lymphoma cell line GUMBUS with photofrin and immediate illumination caused concentration-dependent apoptosis induction in the mode of a plateau curve (Fig. 3A and B). Addition of inhibitors 15 min before and 30 min after photofrin allowed to differentiate between early effects that were dependent on singlet oxygen, NO and peroxynitrite, and subsequent NO/peroxynitrite signaling at lower photofrin concentrations (dependent on superoxide anions, NO, peroxynitrite, hydroxyl radicals, but independent of HOCl) and HOCl signaling at higher photofrin concentrations (dependent on superoxide anions, HOCl, hydroxyl radicals but not NO or peroxynitrite). Addition of caspase-8 inhibitor prior to photofrin caused inhibition of apoptosis in the lower, but not in the higher concentration range of photofrin. In contrast, addition of caspase-8 inhibitor 30 min after photofrin had no more inhibitory effect (Fig. 3C). This is contrasted by the effect of caspase-3 and caspase-9 inhibitors, that completely blocked photofrin-mediated apoptosis induction even if they were added 30 min after photofrin (Fig. 3D). These findings point to an early effect of caspase-8, potentially not directly related to apoptosis execution, and late apoptosis executing effects of caspase-3 and caspase-9 as a consequence of intercellular ROS signaling. The modulatory role of caspase-8 rather than its role for the execution of cell death receptor-dependent apoptosis induction under these conditions was further confirmed through gradual removal of singlet oxygen by histidine, which resulted in a decrease in apoptosis induction and an abrupt shift from caspase-8 independency to caspase-8 dependency (Supplementary Fig. 1).

**Fig. 4:**
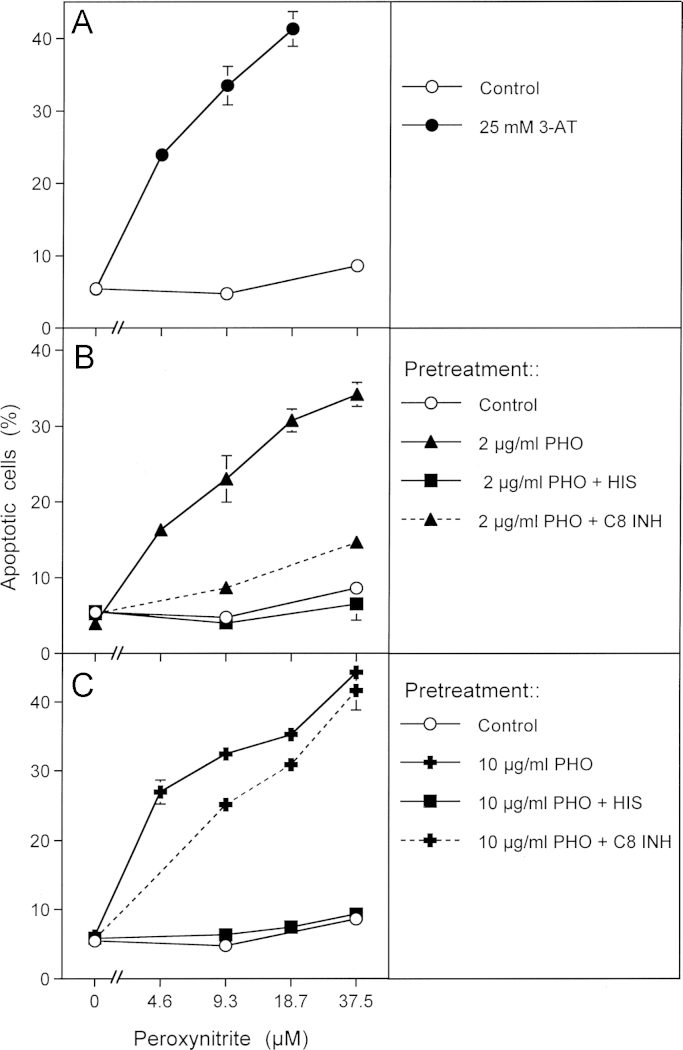
Photofrin-derived singlet oxygen leads to the inactivation of tumor cell protective catalase. (A) Human gastric carcinoma cells MKN-45 (125,000 cells/ml) were incubated for 30 min at room temperature and then were centrifuged, resuspended in fresh medium, centrifuged again and resuspended in fresh medium at a density of 40,000 cells/ml. The assays received either no addition (control) or 25 mM 3-AT. Peroxynitrite was added at the indicated concentrations and the percentages of apoptotic cells were determined after 1.5 h. (B, C) 125,000 MKN-45 cells per ml received either no addition (control) or 2 μg/ml (B) or 10 mg/ml photofrin (C). In addition, 2 μg/ml and 10 μg/ml photofrin were combined with 2 mM histidine (“+HIS”) or 25 mM caspase-8 inhibitor (“+ C8 INH”). The assays were illuminated for 30 min at room temperature, centrifuged, washed twice through addition of fresh medium and centrifugation and then were resuspended at a density of 40,000 cells/ml (100 µl/assay). The assays received the indicated concentrations of peroxynitrite and the percentages of apoptotic cells were determined after additional 1.5 h at 37 °C. Statistical analysis: Apoptosis induction mediated by photofrin after pretreatment with 3-AT or photofrin was highly significant (*p*<0.001). Inhibition by histidine and caspase-8 inhibitor in Fig. 7B and by histidine in Fig. 7C were highly significant (*p*<0.001).

In order to obtain a clear-cut experimental differentiation between singlet oxygen-dependent catalase inactivation and subsequent intercellular ROS-mediated apoptosis signaling, photofrin treatment of tumor cells in the presence of inhibitors of interest was directly followed by a challenge with exogenous peroxynitrite. In this experimental setup, apoptosis induction was independent of intercellular ROS signaling, but was driven by the apoptosis-inducing effect of added peroxynitrite that is counteracted by the peroxynitrite-decomposing potential of membrane-associated catalase [56]. Apoptosis induction by exogenous peroxynitrite was therefore indicative of inactivation of membrane-associated catalase. As shown in Fig. 4A, this protective function was abrogated by the catalase inhibitor 3-AT. Pretreatment with 2 or 10 µg/ml photofrin caused the same degree of catalase inactivation as 3-AT. The photofrin-dependent reaction was completely dependent on singlet oxygen, as seen by the strong inhibition by histidine. Inactivation of catalase by 2 µg/ml photofrin was strongly dependent on the activity of caspase-8, whereas inactivation by 10 µg/ml photofrin was largely independent on caspase-8 (Fig. 4B and C).

**Fig. 5:**
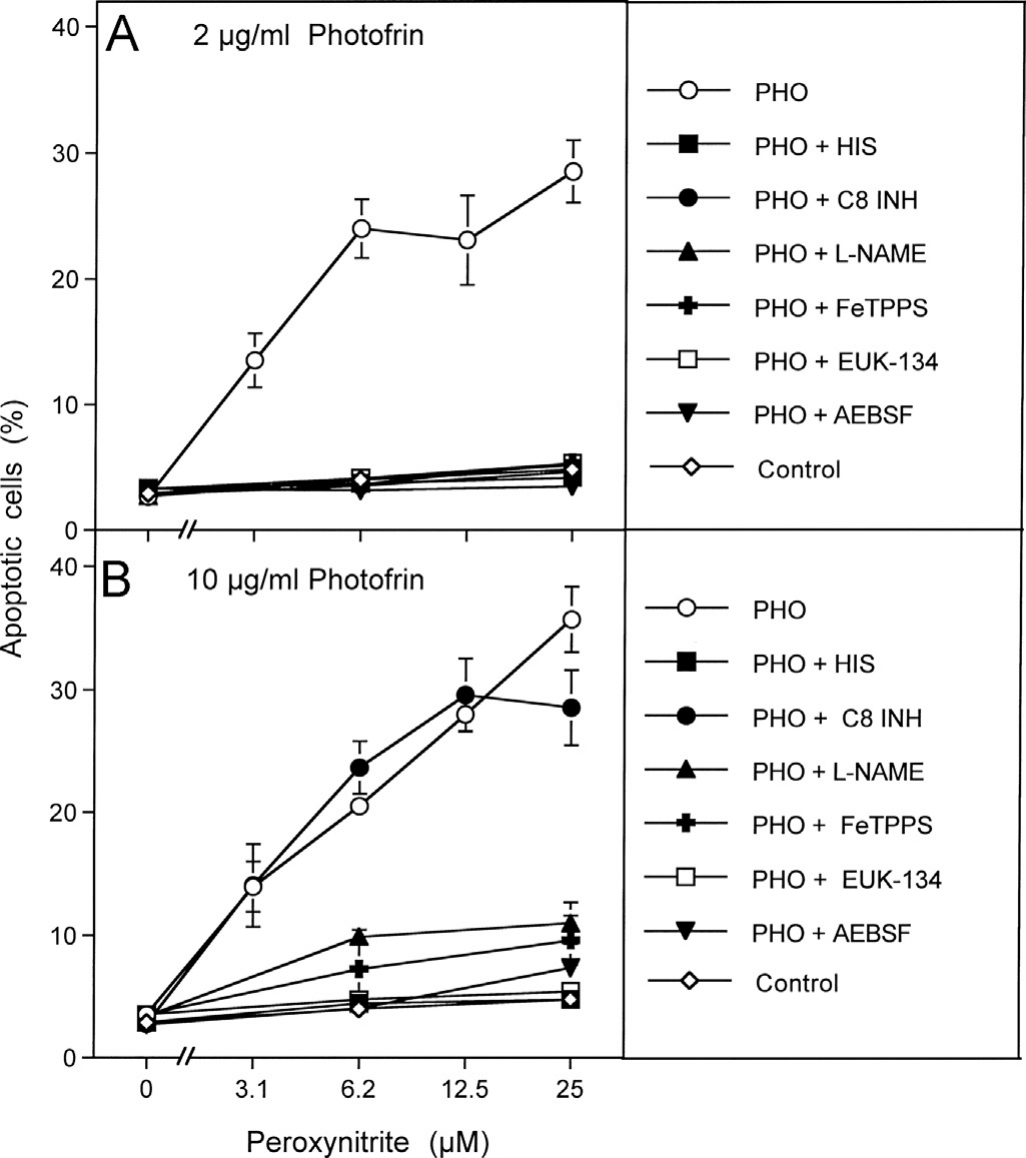
Photofrin-derived singlet oxygen requires generation of cell-derived singlet oxygen for optimal catalase inactivation 125,000 MKN-45 cells per ml received either no photofrin (control) or 2 μg/ml (A) or 10 μg/ml photofrin (B). In addition, as indicated, assays in addition to photofrin contained 2 mM histidine (HIS), 25 μM caspase-8 inhibitor (C8 INH), 2.4 mM l-NAME, 25 mM FeTPPS, 10 μM EUK-134 or 100 μM AEBSF. All assays were illuminated for 30 min at room temperature and then the cells were centrifuged, washed with fresh medium three times and resuspended in fresh medium at a density of 40,000 cells/ml (100 μl/assay). Peroxynitrite was added at the indicated concentration and the percentage of apoptotic cells was determined after 2 h. Statistical analysis: Apoptosis induction by 2 μg/ml and 10 μg/ml, as well as the effects of all inhibitors (except for caspase-8 inhibitor in the presence of 10 μg/ml photofrin) were highly significant (*p*<0.001).

This finding was confirmed in a repeat experiment (Fig. 5) which showed that inactivation of catalase by 2 µg/ml and 10 µg/ml photofrin was dependent on singlet oxygen, as seen by the inhibitory effect of histidine. Again the effect of 2 µg/ml photofrin was dependent on caspase-8, whereas that of 10 µg/ml photofrin was not. Unexpectedly, catalase inactivation by both concentrations of photofrin required ongoing superoxide anion generation, as it was inhibited by the NOX inhibitor AEBSF. The inhibitory effects of EUK-134, l-NAME and FeTPPS point to an involvement of H_2_O_2_, NO and peroxynitrite in photofrin-mediated catalase inactivation. If these conclusions on autoamplification of singlet oxygen generation were correct, tumor cells that had been pretreated with exogenous singlet oxygen should cause inactivation of membrane-associated catalase in neighboring untreated cells through their own generation of singlet oxygen.

**Fig. 6:**
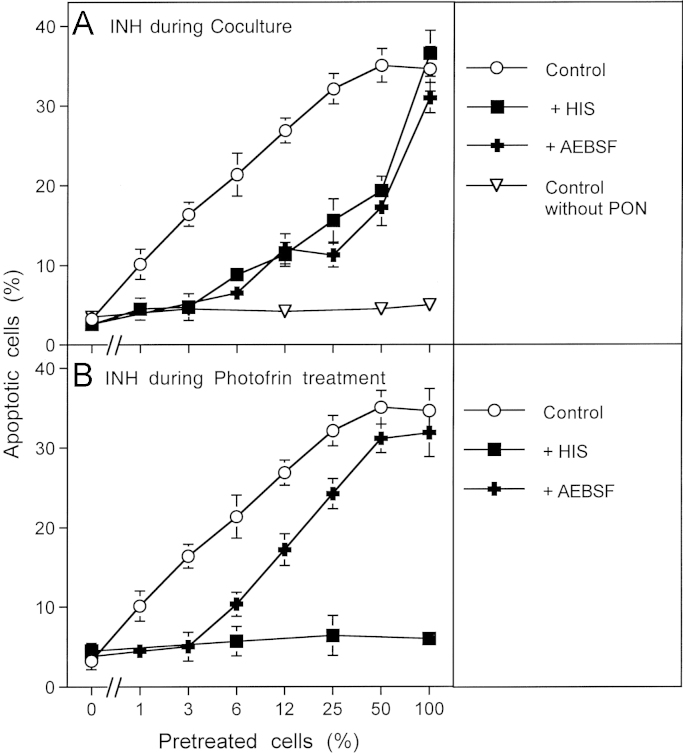
Functional proof for the generation of cell-derived singlet oxygen by tumor cells pretreated with exogenous singlet oxygen MKN-45 cells (125,000 cells/ml) received 10 μg/ml photofrin and were illuminated with visible light for 20 min. Cells were centrifuged and resuspended in excess new medium. This washing step for the removal of photofrin was repeated twice. Photofrin-pretreated cells were mixed with untreated cells at the ratios indicated in the abscissa and were incubated at 37 °C for 20 min. AEBSF was added to a final concentration of 100 μM and 200 μM peroxynitrite (PON) was added (“Control”). Control assays without PON were prepared in parallel. As indicated in the figure, some assays contained 2 mM histidine (HIS) or 100 μM AEBSF either during the coculture (A) or during pretreatment with photofrin (B). The percentages of apoptotic cells were determined after 1.5 h. Statistical analysis: (A) The effect of 3–100% pretreated cells on catalase inactivation, the apoptosis-inducing effect of peroxynitrite and the inhibitory effect of histidine and AEBSF between 3% and 50% pretreated cells were highly significant (*p*<0.001). (B) The inhibitory effect of histidine and the inhibitory effect of AEBSF for 3% pretreated cells were highly significant (*p*<0.001).

Therefore, MKN-45 tumor cells were pretreated with 10 µg/ml photofrin and illuminated for 20 min to allow singlet oxygen generation. Photofrin was then removed through several washing steps and the cells were mixed at increasing percentages with untreated MKN-45 cells and incubated for 20 min. The degree of catalase inactivation was then determined through a challenge with exogenous peroxynitrite. As shown in Fig. 6, addition of photofrin-pretreated cells to untreated cells caused a sensitization for the apoptosis-inducing effect of peroxynitrite that increased with the percentage of pretreated cells, but was higher than to be expected from the ratio of pretreated cells. For example, 6% pretreated cells already caused half-maximal induction of apoptosis when compared to a population of 100% pretreated cells. When either histidine or AEBSF had been added during the coculture phase, apoptosis induction by peroxynitrite correlated precisely with the percentage of pretreated cells (Fig. 6A). This finding demonstrates that the pretreated cells must have generated cell-derived singlet oxygen that triggered catalase inactivation in neighboring control cells. Superoxide anion generation by active NOX was necessary for this reaction. When the singlet oxygen scavenger histidine had been present during pretreatment of the cells with illuminated photofrin (Fig. 6B), the pretreated cell population as well as the cocultures remained insensitive towards peroxynitrite, demonstrating the necessity for initial singlet oxygen formation. The presence of AEBSF during the pretreatment step seemed to restrict the action of photofrin-derived singlet oxygen to direct catalase inactivation and prevented amplification of singlet oxygen generation during this phase.

Singlet oxygen-dependent inactivation of tumor cell protective catalase was sufficient to explain the onset of subsequent ROS-mediated apoptosis signaling, as addition of exogenous catalase directly after illumination of MKN-45 tumor cells in the presence of photofrin prevented apoptosis induction (Supplementary Fig. 2). In order to determine whether the caspase-8-dependent step of catalase inactivation at lower concentrations of photofrin was controlled by the FAS receptor, MKN-45 cells transfected with control siRNA (siCo) or siRNA directed against the FAS receptor (siFASR) were treated with increasing concentrations of photofrin and apoptosis induction was compared to photofrin-mediated apoptosis induction in MKN-45 cells in the absence and presence of caspase-8 inhibitor. As shown in Supplementary Fig. 3, siRNA-mediated knockdown of FAS receptor activity caused the same pattern of inhibition of photofrin-mediated apoptosis induction as inhibition of caspase-8, pointing to a central and dominant role of the FAS receptor for the caspase-8-dependent effect during apoptosis induction mediated by low concentrations of photofrin.

**Fig. 7:**
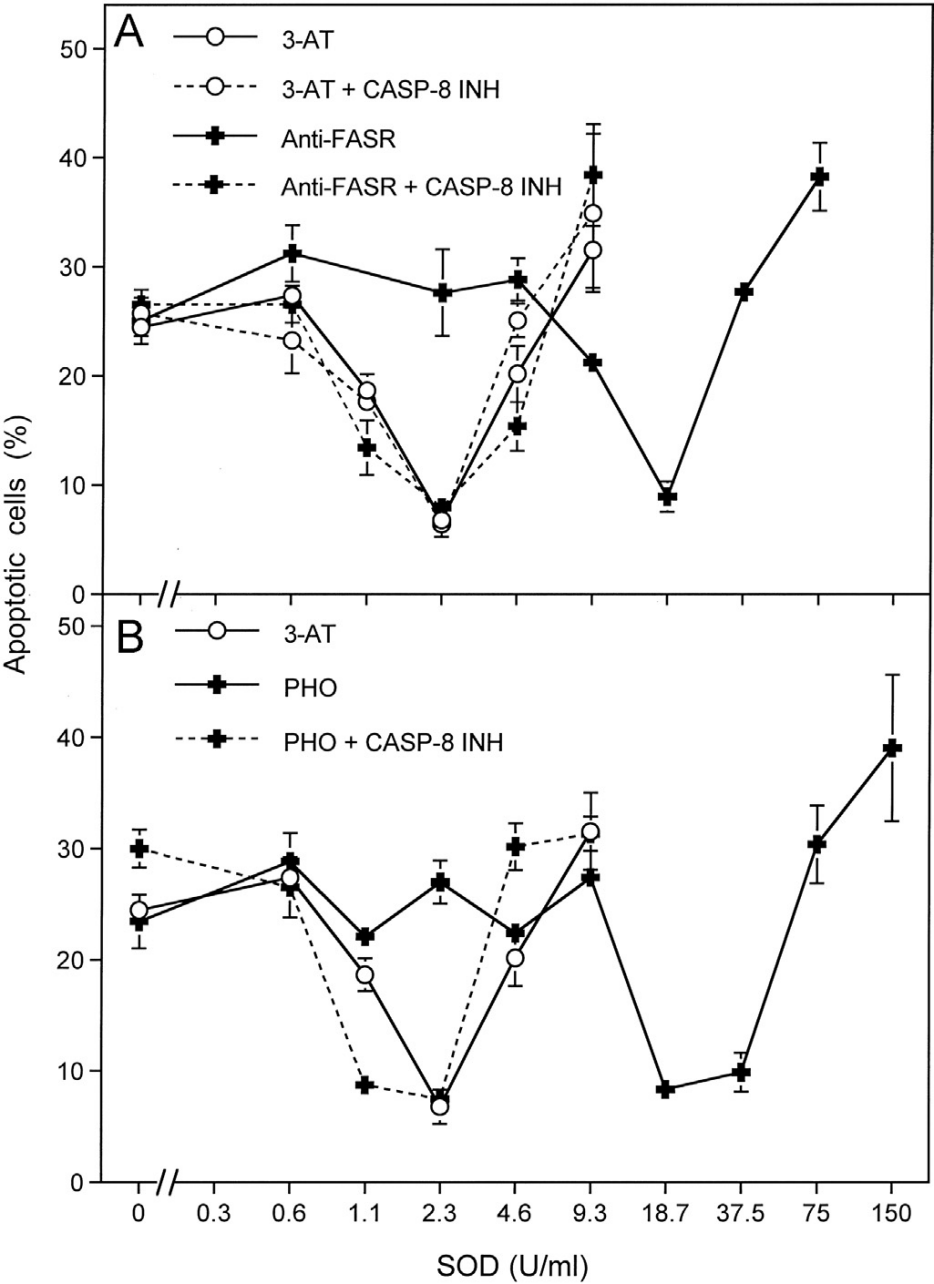
Activation of the FAS receptor by specific antibodies or by photofrin-derived singlet oxygen causes enhancement of extracellular superoxide anion production. (A) 12,500 MKN-45 cells per assay received 100 mM 3-AT (“3-AT”), 100 mM 3-AT plus 25 μM caspase-8 inhibitor (“3-AT+CASP8 INH”), 10 μg/ml antibody directed against the FAS receptor (“Anti-FASR”) or 10 μg/ml antibody directed against the FAS receptor plus 25 mM caspase-8 inhibitor (“Anti-FASR+CASP8 INH”). The assays received the indicated concentrations of Cu/ZnSOD and were incubated for 5 h at 37 °C. (B) 12,500 MKN-45 cells per assay received 1 μg/ml photofrin (“PHO”) or 1 µg/ml photofrin plus 25 μM caspase-8 inhibitor (“PHO+CASP8 INH”). The assays were illuminated with visible light for 30 min and then the indicated concentrations of Cu/ZnSOD were added. The percentages of apoptotic cells were determined after 5 h. For comparison, the assays containing 100 mM 3-AT (as described under (A)) that had been performed in parallel were included into Fig. 7B. Statistical analysis: The effect of antibodies directed against the FAS receptor and of photofrin, as well as the abrogation of these effects by caspase-8 inhibitor were highly significant (*p*<0.001).

The activation of the FAS receptor by singlet oxygen and a functional interconnection between the FAS receptor and superoxide anion production have been recently described [67–69]. We endeavored to verify these effects under the conditions of our cell culture system. Activation of the FAS receptor through binding of specific antibodies directed against the receptor caused an increase in superoxide anion production of the tumor cells used (Fig. 7A). This increase was dependent on the activity of caspase-8. Generation of extracellular singlet oxygen by photofrin had the same effect as activation of the receptors through antibody binding (Fig. 7B) and was also mediated by caspase-8. Thus, in our cell system, singlet oxygen seems to activate the FAS receptor which leads to caspase-8-dependent stimulation of superoxide anion synthesis.

## 5 Discussion

Our data show that *intracellular* generation of singlet oxygen after illumination of photofrin-loaded cells causes massive cell death (i) without dependence on intercellular signaling, (ii) without an involvement of NOX-derived superoxide anions and (iii) without discrimination between nonmalignant and malignant cells. In contrast, *extracellular* singlet oxygen generation through illumination of *extracellular* photofrin (before its entry into the cells) (i) is dependent on the density of the tumor cells, (ii) is dependent on NOX-dependent extracellular superoxide anion synthesis and (iii) causes apoptosis selectively in tumor cells without affecting nonmalignant cells. The selective action of extracellular singlet oxygen is based on its potential to target membrane-associated catalase of tumor cells [60,61]. It thus provokes subsequent intercellular superoxide anion-controlled apoptosis signaling. Utilization of two characteristic and specific ROS-related features of tumor cell, namely NOX1-dependent generation of extracellular superoxide anions and membrane-associated catalase, warrants strict selectivity of apoptosis induction with respect to the tumor phenotype.

As nontransformed cells lack sustained generation of superoxide anions by NOX1, and to not express extracellular catalase [23,26,29,30,56], they are not affected by singlet oxygen in the concentration range applied in our experiments. Therefore, when NOX1, that discriminates malignant cells from nonmalignant cells, is inhibited in tumor cells, they loose their sensitivity for apoptosis induction mediated by singlet oxygen.

**Fig. 8:**
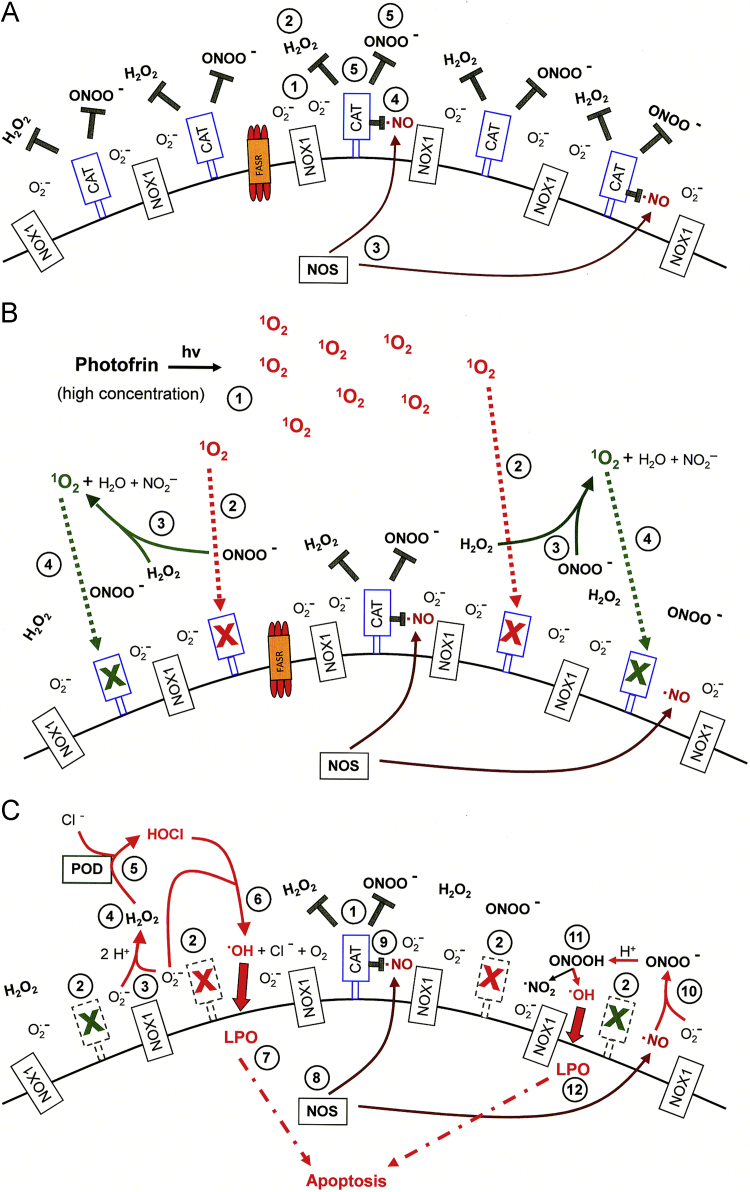
High concentrations of photofrin-derived singlet oxygen triggers the generation of cell-derived singlet oxygen, catalase inactivation and intercellular ROS-dependent apoptosis signaling through the NO/peroxynitrite and HOCl signaling pathway. (A) Tumor cells have activated NOX1 that generates extracellular superoxide anions (#1). The resultant H_2_O_2_ (#2) is decomposed by membrane-associated catalase and thus HOCl signaling is prevented. NO synthase (NOS) generates NO (#3) which is either oxidated by membrane-associated catalase (#4) or reacts with superoxide anions and forms peroxynitrite (#5) that is decomposed by catalase. Oxidation of NO and decomposition of peroxynitrite by catalase prevent NO/peroxynitrite signaling. (B) Singlet oxygen derived from high concentrations of illuminated photofrin (#1) inactivates a substantial number of catalase molecules on the surface of the tumor cell (#2). Thus, H_2_O_2_ and peroxynitrite are not decomposed locally and form singlet oxygen (#3) which inactivates further catalase molecules (#4). (C) As a result of (B), the surface of the tumor cell carries a sufficiently high number of inactivated catalase molecules (#2) besides residual active catalase (#1). As a consequence, superoxide anions derived from NOX1 (#3) dismutate and form H_2_O_2_ (#4) that is no longer decomposed by catalase and drives peroxidase (POD)-catalyzed HOCl synthesis (#5). HOCl reacts with superoxide anions (#6), thereby generating hydroxyl radicals that cause lipid peroxidation (#7) and subsequent apoptosis induction through the mitochondrial pathway of apoptosis. NO synthase (NOS) generates NO (#8) that is not oxidated by inactived catalase (#9) and forms peroxynitrite after its reaction with superoxide anions (#10). As peroxynitrite is not decomposed, protonation of peroxynitrite results in the formation peroxynitrous acid (#11) that generates NO_2_ and hydroxyl radicals that cause lipid peroxidation (LPO) and apoptosis induction (#12).

**Fig. 9:**
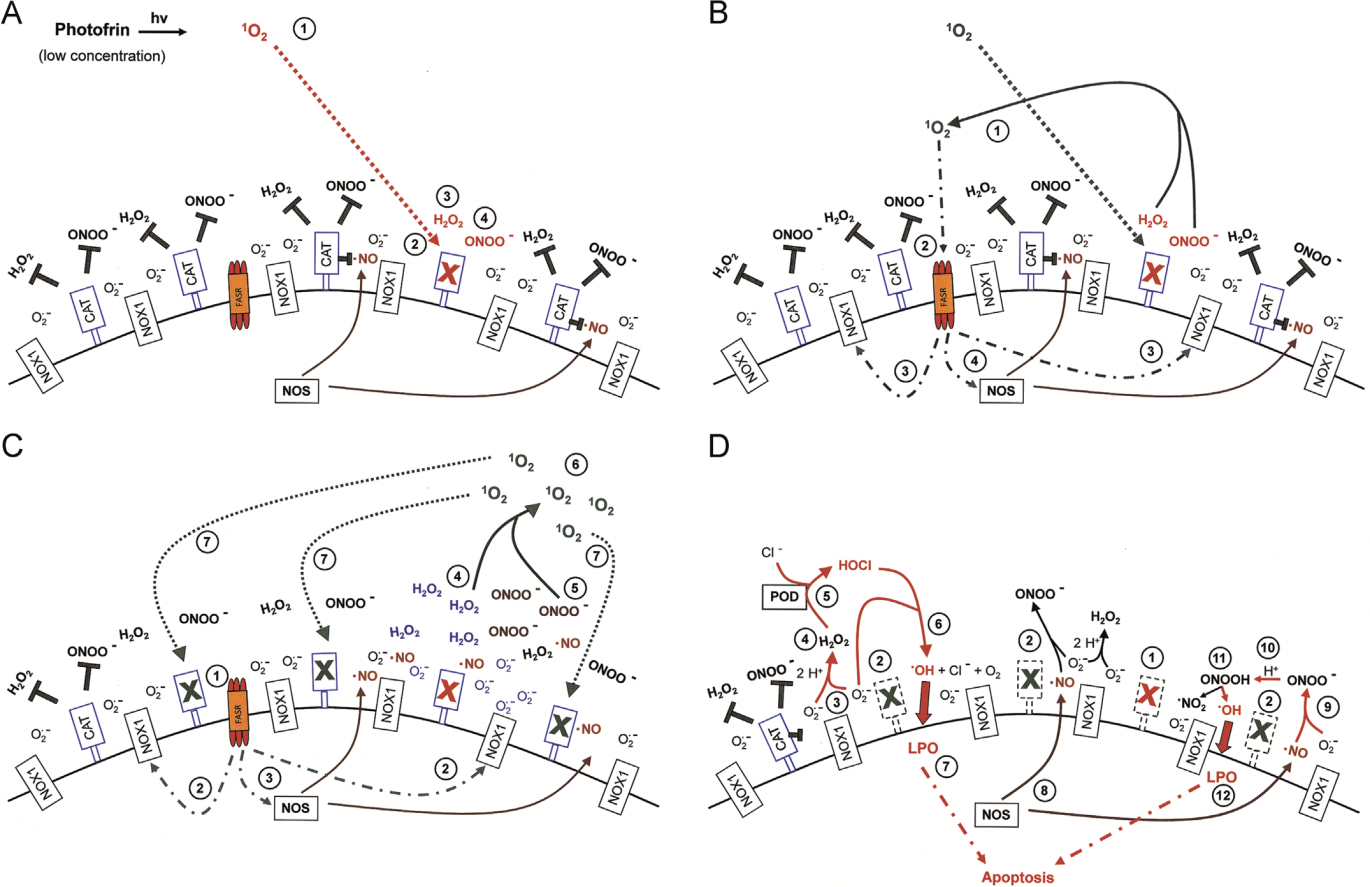
Catalase inactivation by low concentrations of photofrin-derived singlet oxygen requires an additional amplification step that is mediated by the FAS receptor and caspase-8A. (A) Low concentrations of singlet oxygen generated by low concentrations of illuminated photofrin (#1) inactivate few catalase molecules on the surface of tumor cells (#2). This allows a local stabilization of H_2_O_2_ (#3) and peroxynitrite (#4). (B) Locally stabilized H_2_O_2_ and peroxynitrite form singlet oxygen (#1) that activates the FAS receptor in a ligand-independent mode (#2). As a consequence, NOX1 activity is enhanced (#3) and NOS is induced (#4). (C) As a consequence of FAS receptor activation by singlet oxygen (#1), activation of NOX (#2) and induction of NOS (#3), more H_2_O_2_ (#4) and more peroxynitrite (#5) is generated. This allows for the formation of more singlet oxygen (#6) and inactivation of additional catalase molecules (#7). (D) As a consequence of primary (#1) and secondary inactivation of catalase (#2), H_2_O_2_ generated through dismutation of superoxide anions derived from NOX1 (#3) is no longer decomposed (#4) by catalase and drives peroxidase (POD)-catalyzed HOCl synthesis (#5). HOCl reacts with superoxide anions (#6), thereby generating hydroxyl radicals that cause lipid peroxidation (#7) and subsequent apoptosis induction through the mitochondrial pathway of apoptosis. NO synthase (NOS) generates NO (#8) that is not oxidated by inactived catalase (#9) and forms peroxynitrite after its reaction with superoxide anions (#10). As peroxynitrite is not decomposed, protonation of peroxynitrite results in the formation peroxynitrous acid (#11) that generates NO_2_ and hydroxyl radicals that cause lipid peroxidation (LPO) and apoptosis induction (#12).

The biochemical mechanism leading to the inactivation of tumor cell protective catalase by exogenous singlet oxygen is substantially more complex than that of direct catalase inhibitors like 3-AT or neutralizing antibodies directed towards catalase [56] as can be seen in Figs. 8 and 9. In tumor cells, extracellular singlet oxygen seems to trigger an initial fast autoamplificatory step that causes the generation of cell-derived extracellular singlet oxygen. This autoamplification of singlet oxygen through versatile usage of tumor cell specific ROS chemistry finally leads to the inactivation of catalase. This is followed by intercellular superoxide anion-controlled ROS-dependent apoptosis signaling of the tumor cells. NOX1-generated extracellular superoxide anions seem to be necessary for both steps. In addition, NO, peroxynitrite and H_2_O_2_ are required for the de novo generation of additional singlet oxygen during this early and fast step. The *extracellular* localization of the newly synthesized singlet oxygen is warranted through the extracellular generation of tumor cell-derived superoxide anions that are required for the generation of H_2_O_2_ and peroxynitrite.

At low concentrations of the initially acting photofrin-derived singlet oxygen, further amplification of ROS/RNS interactions by the FAS receptor mediated through caspase-8 is necessary. Caspase-8 enhances NOX1 activity and thus increases the concentrations of H_2_O_2_ and potentially also of peroxynitrite. In addition, caspase-8-dependent induction of NOS expression [70] might contribute to enhanced peroxynitrite and singlet oxygen generation, but has not yet been demonstrated in this system. Inactivation of protective catalase seems to be sufficient to explain these initial effects as (i) subsequent intercellular ROS-dependent apoptosis signaling cannot occur in the presence of active membrane-associated catalase and as (ii) the apoptosis-mediating effect of photofrin-derived singlet oxygen is completely abrogated through the addition of exogenously added catalase and as (iii) catalase inactivation after photofrin treatment can be directly demonstrated through challenging photofrin-treated tumor cells with exogenous peroxynitrite. The strength and the quality of intercellular ROS signaling that follows photofrin-mediated catalase inactivation depend on the initial concentration of photofrin-derived singlet oxygen [56,63].

Figs. 8 and 9 summarize the mechanistic details of the action and consequences of high and low concentrations of photofrin-derived singlet oxygen for the survival of tumor cells. Fig. 8A shows the surface of a NOX1-expressing tumor cell that is protected against intercellular apoptosis-inducing ROS signaling as membrane-associated catalase interferes (i) with NO/peroxynitrite signaling through oxidation of NO and decomposition of peroxynitrite and (ii) with HOCl signaling through decomposition of H_2_O_2_, the substrate of peroxidase-dependent HOCl synthesis. After inactivation of a substantial subpopulation of catalase molecules by a high concentration of photofrin-derived singlet oxygen (Fig. 8B), the generation of H_2_O_2_ and peroxynitrite is no longer outbalanced at the site of inactivated catalase and singlet oxygen is generated through the interaction between H_2_O_2_ and peroxynitrite [62]. (See Supplementary Figs. 4–6 for details.) As a consequence, more membrane-associated catalase molecules are inactivated and thus optimal intercellular ROS signaling is established (Fig. 8C). The necessity for the second round of singlet oxygen generation from cell-derived H_2_O_2_ and peroxynitrite is proven by the inhibitory effect of AEBSF, l-NAME, EUK-138 and FeTPPS on catalase inactivation by high concentrations of photofrin. A direct apoptosis-inducing effect of singlet oxygen in the concentration range of photofrin applied here can be excluded, as there was no significant apoptosis inducing effect when intercellular ROS-driven signaling was inhibited. A strong inactivation of catalase established a sufficiently high concentration of H_2_O_2_ for peroxidase-dependent HOCl signaling, but also counteracted NO/peroxynitrite signaling, as recently described [56].

When low concentrations of photofrin are applied, the initial direct inactivation of catalase is not sufficient to allow optimal singlet oxygen generation from cell-derived H_2_O_2_ and peroxynitrite and a strong inactivating effect on catalase. (Fig. 9A). Therefore, an additional amplification step seems to be required. It is based on the activation of the FAS receptor by singlet oxygen and the subsequent caspase-8 dependent stimulation of NOX1 activity (Fig. 9B). As a consequence, the formation of H_2_O_2_ and peroxynitrite seem to be enhanced and the resultant increased production of singlet oxygen is sufficiently high to inactivate catalase at least to a degree that allows for the establishment of intercellular NO/peroxynitrite signaling (Fig. 9C).

An additional direct indication for cellular singlet oxygen generation after initial treatment of tumor cells with exogenous singlet oxygen was established through coculture experiments with photofrin-pretreated and untreated tumor cells. These experiments (Fig. 6) showed that photofrin-pretreated cells caused singlet oxygen-dependent inactivation of catalase in neighboring untreated cells.

The amplificatory role of the FAS receptor and caspase-8 for singlet oxygen generation through H_2_O_2_/peroxynitrite interaction has been deduced from published work [67–70] and has been directly confirmed in the experiments shown in Supplementary Fig. 3 and in Fig. 7. As knockdown of the FAS receptor resulted in an identical effect as inhibition of caspase-8, the functional connection between the FAS receptor and caspase-8 is sufficient to explain the amplificatory mechanism during singlet oxygen-dependent stimulation of singlet oxygen generation. It is in line with work published by other groups [67–70] and has also been shown to be relevant for cyanidin and 4-hydroxynonenal-mediated induction of apoptosis in tumor cells [63,64]. As the FAS receptor/caspase-8 system was dispensable after stimulation of NOX1 through TGF-beta, caspase-8-dependent activation of NOX1 seems to be the essential step in the scenario described here and caspase-8-dependent activation of NOS seems to be less relevant [63,64]. As the effect of caspase-8 is restricted to the time of inactivation of protective catalase and as it is only required when the initial concentration of singlet oxygen is very low, the role of caspase-8 for the amplification of singlet oxygen is confirmed, whereas there is no indication for a direct involvement of caspase-8 in the execution of cell death. The clear dissection of this rather complex multistep scenario is only possible in tumor cells like MKN-45 cells that have been found not to express sufficient FAS receptor for the direct execution of apoptotic cell death through the classical FAS receptor triggered pathway (Bauer, unpublished result). Work in progress (Bauer, unpublished) shows that treatment of tumor cells with a substantially higher concentration of FAS receptor causes the direct activation of the FAS receptor mediated apoptosis pathway by singlet oxygen in parallel to amplification of singlet oxygen-dependent singlet oxygen generation, catalase inactivation and intercellular ROS-mediated apoptosis signaling.

The model experiments presented in this manuscript allow to conclude that singlet oxygen that is generated from cellular sources after modulation of the cellular NO concentration has the potential to drive the biochemical pathways that amplify cellular singlet oxygen generation and finally lead to catalase inactivation and the onset of intercellular ROS-dependent apoptosis signaling in tumor cells. They also demonstrate the complexity of this autoamplificatory system and thus open the path for further modulatory optimization. This may lead to the establishment of novel approaches for tumor therapy that utilize either the generation of cellular singlet oxygen through modulation of the NO metabolism or that are based on optimized photosensitizers that reach the tumor *but do not enter* the tumor cells and thus target specifically tumor cell protective catalase. Both options for potential ROS-based tumor therapy are exciting and promising, as they exploit specific and defined features of tumor cells for their selective destruction.

## Footnotes

Appendix A Supplementary data associated with this article can be found in the online version at doi:10.1016/j.redox.2015.07.006.

## References

[1] Bauer G., Chatgilialoglu C., Gebicki J.L., Gebicka L., Gescheidt G., Golding B.T., Goldstein S., Kaizer J., Merenyi G., Speier G., Wardman P. (2008). Biologically relevant small radicals. Chimia.

[2] Graves D.B. (2012). The emerging role of reactive oxygen and nitrogen species in redox biology and some implications for plasma applications to medicine and biology. J. Phys. D: Appl. Phys..

[3] Irani K., Xia Y., Zweier J.L., Sollott S.J., Der C.J., Fearon E.R., Sundaresan M., Finkel T., Goldschmidt-Clermont P.J. (1997). Mitogenic signalling by oxidants in *Ras*-transformed fibroblasts. Science.

[4] Irani K., Goldschmidt-Clermont P.J. (1998). Ras, superoxide and signal transduction. Biochem. Pharmacol..

[5] Suh Y.-A., Arnold R.S., Lassegue B., Shi J., Xu X., Sorescu D., Chung A.B., Griendling K.K., Lambeth J.D. (1999). Cell transformation by the superoxide-generating oxidase Mox1. Nature.

[6] Yang J.Q., Li S., Domann F.E., Buettner G., Oberley L.W. (1999). Superoxide generation in *v-Ha-ras*-transduced human keratinocyte HaCaT cells. Mol. Carcinog..

[7] Arnold R.S., Shi J., Murad E., Whalen A.M., Sun C.Q., Palavarapu R., Parthasarathy S., Petros J.A., Lambeth J.D. (2001). Hydrogen peroxide mediates the cell growth and transformation caused by the mitogenic oxidase Nox1. Proc. Natl. Acad. Sci. U.S.A..

[8] Mitsushita J., Lambeth J.D., Kamata T. (2004). The superoxide-generating oxidase Nox1 is functionally required for *Ras* oncogenic transformation. Cancer Res..

[9] Tominaga K., Kawahara T., Sano t, Toida K., Kuwano Y., Sasaki H., Kawai T. (2007). Evidence for cancer-associated expression of NADPH oxidase 1 (Nox1)-base oxidase system in the human stomach. Free Radic. Biol. Med..

[10] Laurent E., McCoy J.W., Maccina R.A., Liu W., Cheng G.J., Robine S., Papkoff J., Lambeth J.D. (2008). Nox1 is overexpressed in human colon cancers and correlates with activating mutations in *K-Ras*. Int. J. Cancer.

[11] Ma Q., Cavallin L.E., Yan B., Zhu S., Duran E.M., Wang H., Hala L.P., Dong C., Cesarman E., Mesri E.A., Goldschmidt-Clermont P.J. (2009). Antitumorigenesis of antioxidants in a transgenic Rac1 model of Kaposi's sarcoma. Proc. Natl. Acad. Sci. U.S.A..

[12] Du J., Liu J., Smith B.J., Tsao M.S., Cullen J. (2011). Role of rac-1-dependent NADPH oxidase in the growth of pancreatic cancer. Cancer Gene Ther..

[13] Lopez-Lazaro M. (2007). Excessive superoxide anion generation plays a key role in carcinogenesis. Int. J. Cancer.

[14] Lopez-Lazaro M. (2007). Dual role of hydrogen peroxide in cancer: possible relevance to cancer chemoprevention and therapy. Cancer Lett..

[15] Jürgensmeier J., Schmitt C.P., Viesel E., Höfler P., Bauer G. (1994). TGF-ß-treated normal fibroblasts eliminate transformed fibroblasts by induction of apoptosis. Cancer Res..

[16] Jürgensmeier J., Höfler P., Bauer G. (1994). TGF-ß-induced elimination of transformed fibroblasts by normal cells: independence of cell-to-cell contact and dependence on reactive oxygen species. Int. J. Oncol..

[17] Schaefer D., Jürgensmeier J., Bauer G. (1995). Catechol interferes with TGF-ß-induced elimination of transformed cells by normal cells: implications for the survival of transformed cells during carcinogenesis. Int. J. Cancer.

[18] Langer C., Jürgensmeier J.M., Bauer G. (1996). Reactive oxygen species act both at TGF-ß-dependent and -independent steps during induction of apoptosis of transformed cells by normal cells. Exp. Cell Res..

[19] Hipp M.-L., Bauer G. (1997). Intercellular induction of apoptosis in transformed cells does not depend on p53. Oncogene.

[20] Jürgensmeier J., Bauer G. (1997). Interference of Bcl-2 with intercellular control of carcinogenesis. Int. J. Cancer.

[21] Panse J., Hipp M.-L., Bauer G. (1997). Fibroblasts transformed by chemical carcinogens are sensitive for intercellular induction of apoptosis: implications for the control of oncogenesis. Carcinogenesis.

[22] Beck E., Schäfer R., Bauer G. (1997). Sensitivity of transformed fibroblasts for intercellular induction of apoptosis is determined by their transformed phenotype. Exp. Cell Res..

[23] Engelmann I., Dormann S., Saran M., Bauer G. (2000). Transformed target cell-derived superoxide anions drive apoptosis induction by myeloperoxidase. Redox Rep..

[24] Herdener M., Heigold S., Saran M., Bauer G. (2000). Target cell-derived superoxide anions cause efficiency and selectivity of intercellular induction of apoptosis. Free Radic. Biol. Med..

[25] Schwieger A., Bauer L., Hanusch J., Sers C., Schäfer R., Bauer G. (2001). *Ras* oncogene expression determines sensitivity for intercellular induction of apoptosis. Carcinogenesis.

[26] Heigold S., Sers C., Bechtel W., Ivanovas B., Schäfer R., Bauer G. (2002). Nitric oxide mediates apoptosis induction selectively in transformed fibroblasts compared to nontransformed fibroblasts. Carcinogenesis.

[27] Ivanovas B., Bauer G. (2002). Selective and nonselective apoptosis induction in transformed and nontransformed fibroblasts by exogenous reactive oxygen and nitrogen species. Anticancer Res..

[28] Bauer G. (2000). Reactive oxygen and nitrogen species: efficient, selective and interactive signals during intercellular induction of apoptosis. Anticancer Res..

[29] Bauer G. (2012). Tumor cell protective catalase as a novel target for rational therapeutic approaches based on specific intercellular ROS signaling. Anticancer Res..

[30] Bauer G. (2014). Targeting extracellular ROS signaling of tumor cells. Anticancer Res..

[31] Candeias L.P., Patel K.B., Stratford M.R.L., Wardmann P. (1993). Free hydroxyl radicals are formed on reaction between the neutrophil-derived species superoxide anion and hypochlorous acid. FEBS.

[32] Folkes L.K., Candeias L.P., Wardman P. (1995). Kinetics and mechanisms of hypochlorous acid reactions. Arch. Biochem. Biophys..

[33] Moncada S., Higgs E.A. (1993). The l-arginine-nitric oxide pathway. N. Engl. J. Med..

[34] Moncada S., Higgs E.A., Hodson H.F., Knowles R.G., Lopez-Jaramillo P., McCall T., Palmer R.M.J., Radomski W., Rees D.D., Schulz R. (1991). The l-arginine: nitric oxide pathway. J. Cardiovasc. Pharmacol..

[35] Moncada S., Palmer R.M.J., Higgs E.A. (1991). Nitric oxide: physiology, pathophysiology, and pharmacology. Pharmacol. Rev..

[36] Saran M., Michel C., Bors W. (1990). Reaction of NO with O_2_^−^. Implication for the action of endothelium-derived relaxing factor (EDRF). Free Radic. Res. Commun..

[37] Beckman J.S., Beckman T.W., Chen J., Marshall P.A., Freeman B.A. (1990). Apparent hydroxyl radical production by peroxynitrite: implication for endothelial injury from nitric oxide and superoxide. Proc. Natl. Acad. Sci. U.S.A..

[38] Koppenol W.H., Moreno J.J., Pryor W.A., Ischiropoulos H., Beckman J.S. (1992). Peroxynitrite, a cloaked oxidant formed by nitric oxide and superoxide. Chem. Res. Toxicol..

[39] Huie R.E., Padmaja S. (1993). The reaction of NO with superoxide. Free Radic. Res. Commun..

[40] Goldstein S., Czapski G. (1995). The reaction of.NO with O_2_.^−^ and HO_2_.-: a pulse radiolysis study. Free Radic. Biol. Med..

[41] Gatti R.M., Alvarez B., Vasquez-Vivar J., Radi R., Augusto O. (1998). Formation of spin trap adducts during the decomposition of peroxynitrate. Arch. Biochem. Biophys..

[42] Merényi G., Lind J., Goldstein S., Czapski G. (1998). Peroxynitrous acid homolyzes into .OH and .NO_2_ radicals. Chem. Res. Toxicol..

[43] Goldstein S., Meyerstein D., van Eldik R., Czapski G. (1999). Peroxynitrous acid decomposes via homolysis: evidence from high-pressure pulse radiolysis. J. Phys. Chem. A.

[44] De Milito A., Fais S. (2005). Tumor acidity, chemoresistance and proton pump inhibitors. Future Oncol..

[45] Goldstein S., Czapski G. (1998). Formation of peroxynitrate from the reaction of peroxynitrite with CO_2_: evidence for carbonate radical production. J. Am. Chem. Soc..

[46] Squadrito G.L., Pryor W.A. (1998). Oxidative chemistry of nitric oxide: the roles of superoxide, peroxynitrite, and carbon dioxide. Free Radic. Biol. Med..

[47] Espey M.G., Miranda K.M., Thomas D.D., Xavier S.A., Citrin D., Vitek M.P., Wink D.A. (2002). A chemical perspective on the interplay between NO, reactive oxygen species, and reactive nitrogen species. Ann. N.Y. Acad. Sci..

[48] Augusto O., Bonini M.G., Amanso A.M., Linares E., Santos C.X., De Menzes S.L. (2002). Nitrogen dioxide and carbonate radical anion: two emerging radicals in biology. Free Radic. Biol. Med..

[49] Deichman G.I., Vendrov E.L. (1986). Characteristics of in vitro transformed cells essential for their in vivo survival, selection and metastatic activity. Int. J. Caner.

[50] Deichman G.I., Kluchareva T.E., Matveeva V.A., Kushlinsky N.E., Bassalyk L.S., Vendrov E.L. (1989). Clustering of discrete cell properties essential for tumorigenicity and metastasis. I. Studies of syrian hamster embryo fibroblasts spontaneously transformed in vitro. Int. J. Cancer.

[51] Deichman G., Matveeva V.A., Kashkina L.M., Dyakova N.A., Uvarova E.N., Nikiforov M.A., Gudkov A.V. (1998). Cell transforming genes and tumor progression *in vivo* unified secondary phenotypic cell changes. Int. J. Cancer.

[52] Deichman G. (2000). Natural selection and early changes of phenotype of tumor cells *in vivo*: acquisition of new defense mechanisms. Biochemistry (Mosc.).

[53] Deichman G. (2002). Early phenotypic changes of in vitro transformed cells during in vivo progression: possible role of the host innate immunity. Semin. Cancer Biol..

[54] Bechtel W., Bauer G. (2009). Catalase protects tumor cells against apoptosis induction by intercellular ROS signaling. Anticancer Res..

[55] Bechtel W., Bauer G. (2009). Modulation of intercellular ROS signaling of human tumor cells. Anticancer Res..

[56] Heinzelmann S., Bauer G. (2010). Multiple protective functions of catalase against intercellular apoptosis-inducing ROS signaling of human tumor cells. Biol. Chem..

[57] Brunelli L., Yermilov V., Beckman J.S. (2001). Modulation of catalase peroxidatic and catalytic activity by nitric oxide. Free Radic. Biol. Med..

[58] Gebicka L., Didil J. (2009). Catalytic scavenging of peroxynitrite by catalase. Int. J. Inorg. Biochem..

[59] Castano A.P., Demidova T.N., Hamblin M.R. (2005). Mechanism in photodynamic therapy: part two-cellular signaling, cell metabolism and modes of cell death. Photodyn. Ther..

[60] Escobar J.A., Rubio A., Lissi E.A. (1996). SOD and catalase inactivation by singlet oxygen and peroxyl radicals. Free Radic. Biol. Med..

[61] Kim Y.K., Kwon O.J., Park J.-W. (2001). Inactivation of catalase and superoxide dismutase by singlet oxygen derived from photoactivated dye. Biochimie.

[62] Di Mascio P., Bechara E.J.H., Medeiros M.H.G., Briviba K., Sies H. (1994). Singlet molecular oxygen production in the reaction of peroxynitrite with hydrogen peroxide. FEBS Lett..

[63] Scheit K., Bauer G. (2015). Direct and indirect inactivation of tumor cell protective catalase by salicylic acid and anthocyanidins reactivates intercellular ROS signaling and allows for synergistic effects. Carcinogenesis.

[64] Bauer G., Zarkovic N. (2015). Revealing mechanisms of selective, concentration-dependent potentials of 4-hydroxy-2-nonenal to induce apoptosis in cancer cells through inactivation of membrane-associated catalase. Free Radic. Biol. Med..

[65] Alvarez B., Denicola A., Radi R. (1995). Reaction between peroxynitrite and hydrogen peroxide: formation of oxygen and slowing of peroxynitrite decomposition. Chem. Res. Toxicol..

[66] Badway J.A., Karnovsky M.L. (1980). Active oxygen species and the functions of phagocytic leukocytes. Annu. Rev. Biochem..

[67] Zhuang S., Demir J.T., Kochevar I.E. (2001). Protein kinase C inhibits singlet oxygen-induced apoptosis by decreasing caspase-8 activation. Oncogene.

[68] Suzuki Y., Ono Y., Hirabayashi Y. (1998). Rapid and specific reactive oxygen species generation via NADPH oxidase activation during FAS-mediated apoptosis. FEBS Lett..

[69] Reinehr R., Becker S., Eberle A., Grether-Beck S., Häussinger D. (2005). Involvement of NADPH oxidase isoforms and src family kinases in CD95-dependent hepatocyte apoptosis. J. Biol. Chem..

[70] Selleri C., Sato T., Raiola A.M., Rotoli B., Young N.S., Maciejewski J.P. (1997). Induction of nitric oxide synthase is involved in the mechanism of FAS-mediated apoptosis in hematopoietic cells. Br. J. Hematol..

[71] Temme J., Bauer G. (2013). Low-dose gamma irradiation enhances superoxide anion production by nonirradiated cells through TGF-β1-dependent bystander signaling. Rad. Res..

[72] Ophoven S.J., Bauer G. (2010). Salen-manganese complexes: sophisticated tools for the study of intercellular ROS signaling. Anticancer Res..

[73] Kerr J.F.R., Wyllie A.H., Currie A.R. (1972). Apoptosis: a basic biological phenomenon with wide-ranging implications in tissue kinetics. Br. J. Cancer.

[74] Elmore S. (2007). Apoptosis: a review of programmed cell death. Toxicol. Pathol..

[75] Bauer G., Bereswill S., Aichele P., Glocker E. (2014). *Helicobacter pylori* protects protects oncogenically transformed cells from reactive oxygen species-mediated intercellular induction of apoptosis. Carcinogenesis.

