# Supplementary material for: Singlet oxygen treatment of tumor cells triggers extracellular singlet oxygen generation, catalase inactivation and reactivation of intercellular apoptosis-inducing signaling
Source: Redox Biol. 2015 Jul 17;6:157–68. doi: 10.1016/j.redox.2015.07.006 (PMC4532730; doi:10.1016/j.redox.2015.07.006)
Supplement: Supplementary file 1 — Supplementary materials [file mmc1.doc]

Riethmüller M, Burger N and Bauer G

**Supplementary Material**

**Caspase-8 activity is only required at low initial concentrations of photofrin-derived singlet oxygen**

Treatment of GUMBUS cells with 8 µg/ml photofrin and immediate illumination caused strong subsequent apoptosis induction which was completely independent of caspase-8 (Supplementary Figure 1). When the concentration of singlet oxygen generated by photofrin was gradually lowered by the presence of increasing concentrations of the singlet oxygen scavenger histidine, apoptosis induction declined gradually whereas it abruptly became dependent on caspase-8 activity. This finding shows a necessary role of caspase-8 at lower initial singlet oxygen concentration, an independence of caspase-8 at optimal initial concentrations of singlet oxygen and a rather sharp shift between these two biochemical scenarios. This finding also indicates the modulating role of caspase-8, rather than a role of caspase-8 in the execution of the death receptor-mediaed apoptosis pathway in this context.

*Supplementary Figure 1*


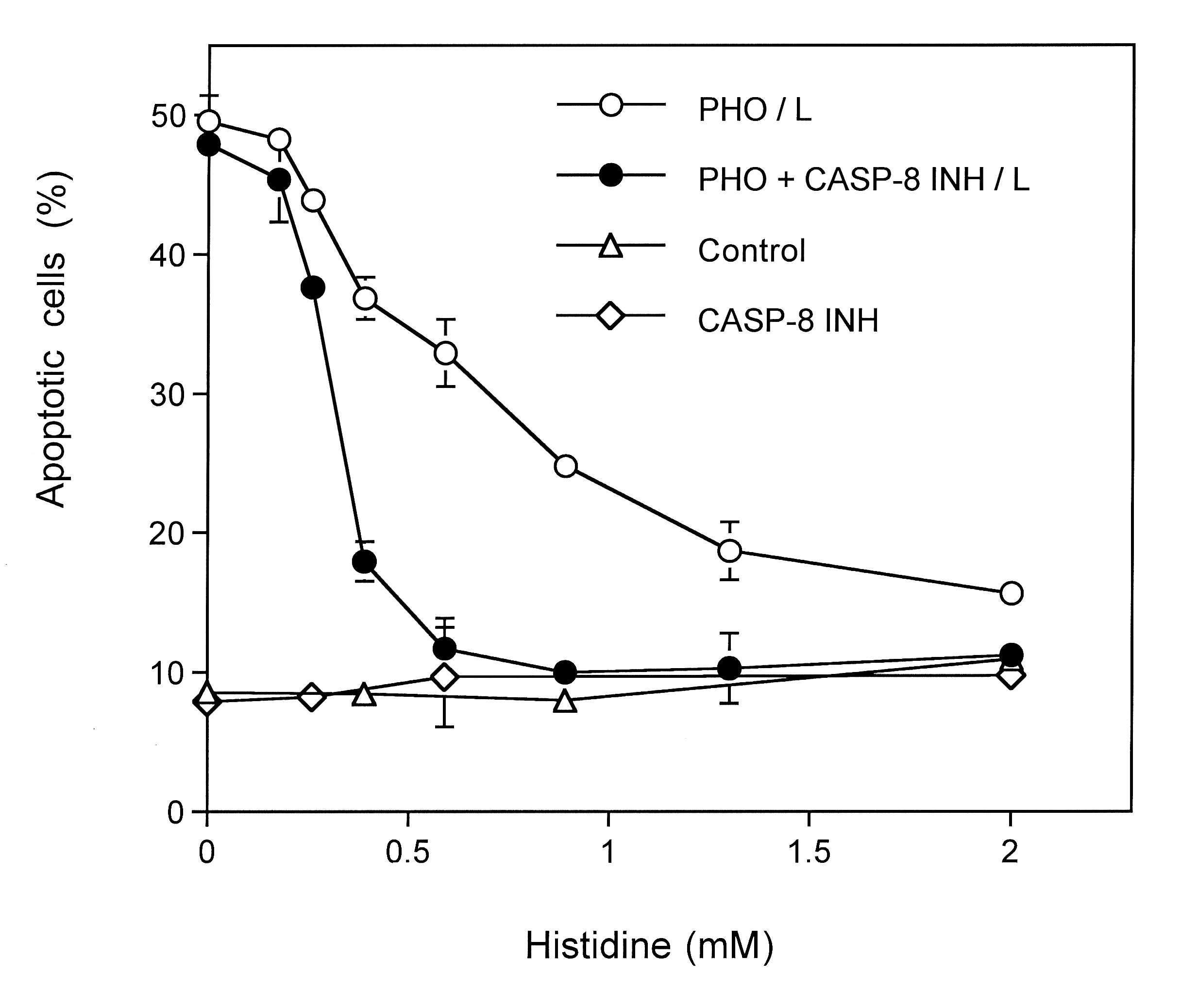


Supplementary Figure 1

**Gradual scavenging of singlet oxygen by histidine causes inhibition of photofrin-mediated apoptosis induction and a shift from caspase-8-independency to caspase-8-dependency**

25 000 GUMBUS cells per assay received either 25 µM caspase-8 inhibitor, 8 µg/ml photofrin, 8µg/ml photofrin plus 25 µM caspase-8-inhibitor or remained free of photofrin and caspase-8 inhibitor (control). All assays received the indicated concentrations of histidine and were then illuminated with visible light for 30 min, followed by incubation at 37 °C for 2.5 h, before the percentages of apoptotic cells were determined.

Statistical analysis: Apoptosis induction by photofrin and the effect of caspase-8 inhibitor on apoptosis induction between 0.4 and 1 mM histidine were highly significant (p<0.001).

**II. Inactivation of catalase by singlet oxygen is sufficient for reactivation of apoptosis-inducing ROS signaling**

To clarify whether singlet oxygen-dependent inactivation of tumor cell protective catalase was sufficient to explain the onset of subsequent ROS-mediated apoptosis signaling, MKN-45 tumor cells were illuminated immediately after addition of photofrin

*Supplementary Figure 2*


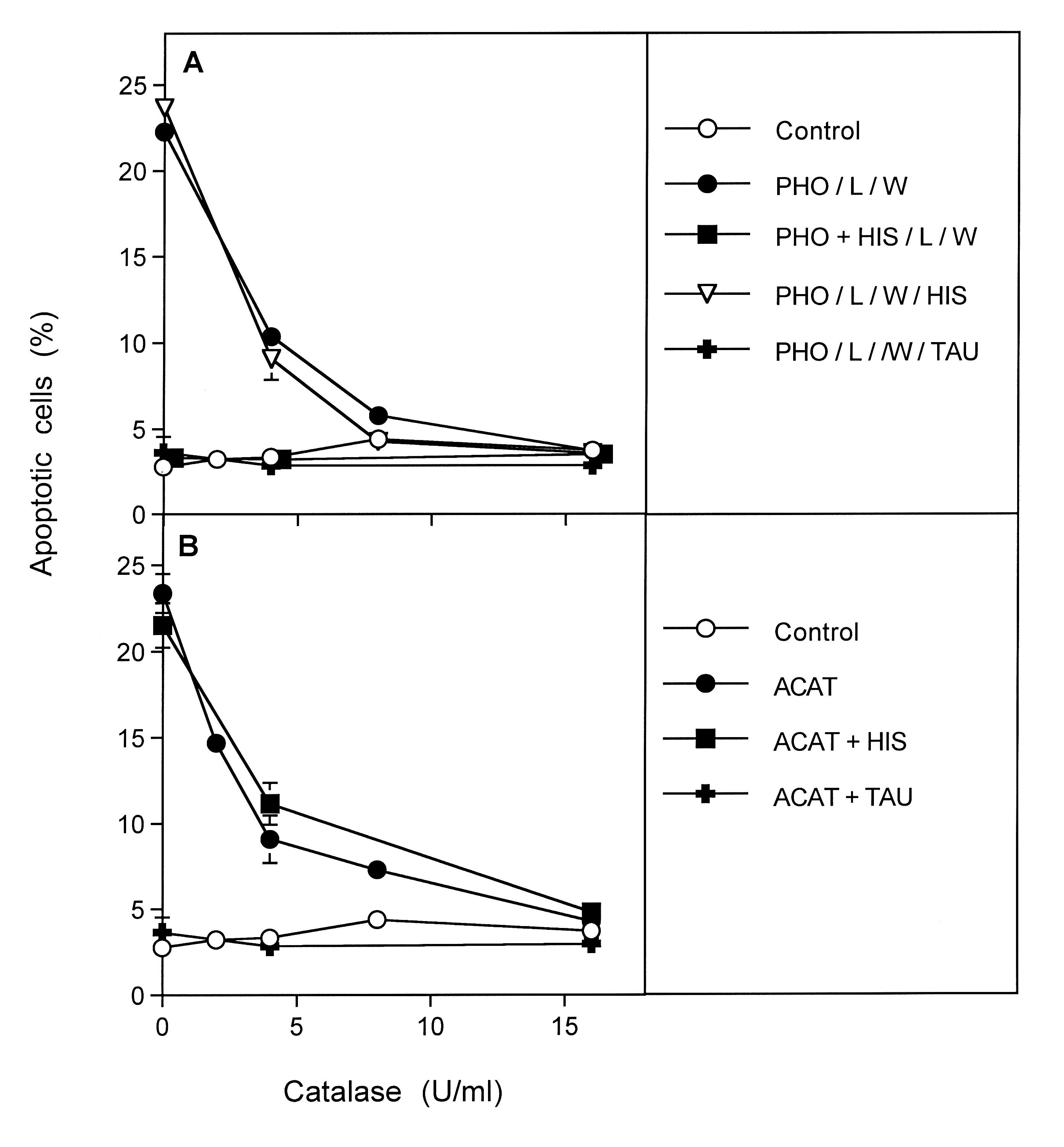


Supplementary Figure 2

**Inactivation of catalase by singlet oxygen is sufficient to explain the reactivation of apoptosis-inducing ROS signaling**

A. 12 500 MKN-45 cells per assay received no addition (control), received 2 µg/ml photofrin, were illuminated for 30 min and washed (“PHO/L/W”), received 2 µg/ml photofrin plus 2 mM histidine, were illuminated for 30 min and washed (“PHO+ HIS/L/W”), received 2 µg/ml photofrin, were illuminated for 30 min and washed and received than 2 mM histidine (“PHO/L/W/HIS”) or received 2 µg/ml photofrin, were illuminated for 30 min and washed and received than 50 mM taurine (“PHO/L/W/TAU”). Bovine liver catalase was then added at the indicated concentrations and the assays were further incubated for 3.5 h before the percentages of apoptotic cells were determined.

B. 12 500 MKN-45 cells were treated with 0.2 µg/ml monoclonal antibody directed against human catalase for 20 min and received then either no further addition (“ACAT”) or 2 mM histidine (“ACAT + HIS”) or 50 mM taurine (“ACAT + TAU”). Control assays with control antibodies directed against laminin were prepared in paralell (“control”). Bovine liver catalase was then added to all assays at the indicated concentrations and the assays were further incubated for 3.5 h before the percentages of apoptotic cells were determined.

Statistical analysis: Apoptosis induction by photofrin and aCAT, inhibition by taurine and the inhibitory effect of catalase were highly significant (p<0.001).

and the effect of exogenously added catalase on reactivated intercellular HOCl signaling was then studied. For functional comparison, tumor cells treated with neutralizing antibodies against catalase were tested in parallel. As shown in Supplementary Figure 2, pretreatment with illuminated photofrin had the same effect as neutralizing antibodies against catalase, as it allowed apoptosis induction through the HOCl signaling pathway. HOCl signaling was demonstrated through the inhibition by the HOCl scavenger taurine. Singlet oxygen and NO did not seem to play a role during intercellular signaling, as neither histidine nor L-NAME caused inhibition of apoptosis induction. Catalase inhibited intercellular HOCl signaling completely, indicating that the effect of illuminated photofrin is sufficiently explained by inactivation of tumor cell catalase .

**III. The FAS receptor and caspase-8 are required for optimal catalase inactivation and reactivation of intercellular ROS-mediated apoptosis signaling triggered by photofrin-derived singlet oxygen.**

In order to determine whether the caspase-8-dependent step of catalase inactivation at lower concentrations of photofrin was controlled by the FAS receptor, MKN-45 cells transfected with control siRNA (siCo) or siRNA directed against the FAS receptor (siFASR) (see Supplementary Materials and Methods for detail) were treated with increasing concentrations of photofrin and apoptosis induction was compared to photofrin-mediated apoptosis induction in MKN-45 cells in the absence and presence of caspase-8 inhibitor. As shown in Supplementary Figure 3, photofrin caused apoptosis induction in a concentration-dependent way and the mode of an optimum curve. The presence of caspase-8 inhibitor prevented apoptosis induction at low concentrations of photofrin and allowed apoptosis induction at high concentrations of photofrin. Thereby the optimum curve was shifted to a plateau-type

*Supplementary Figure 3*


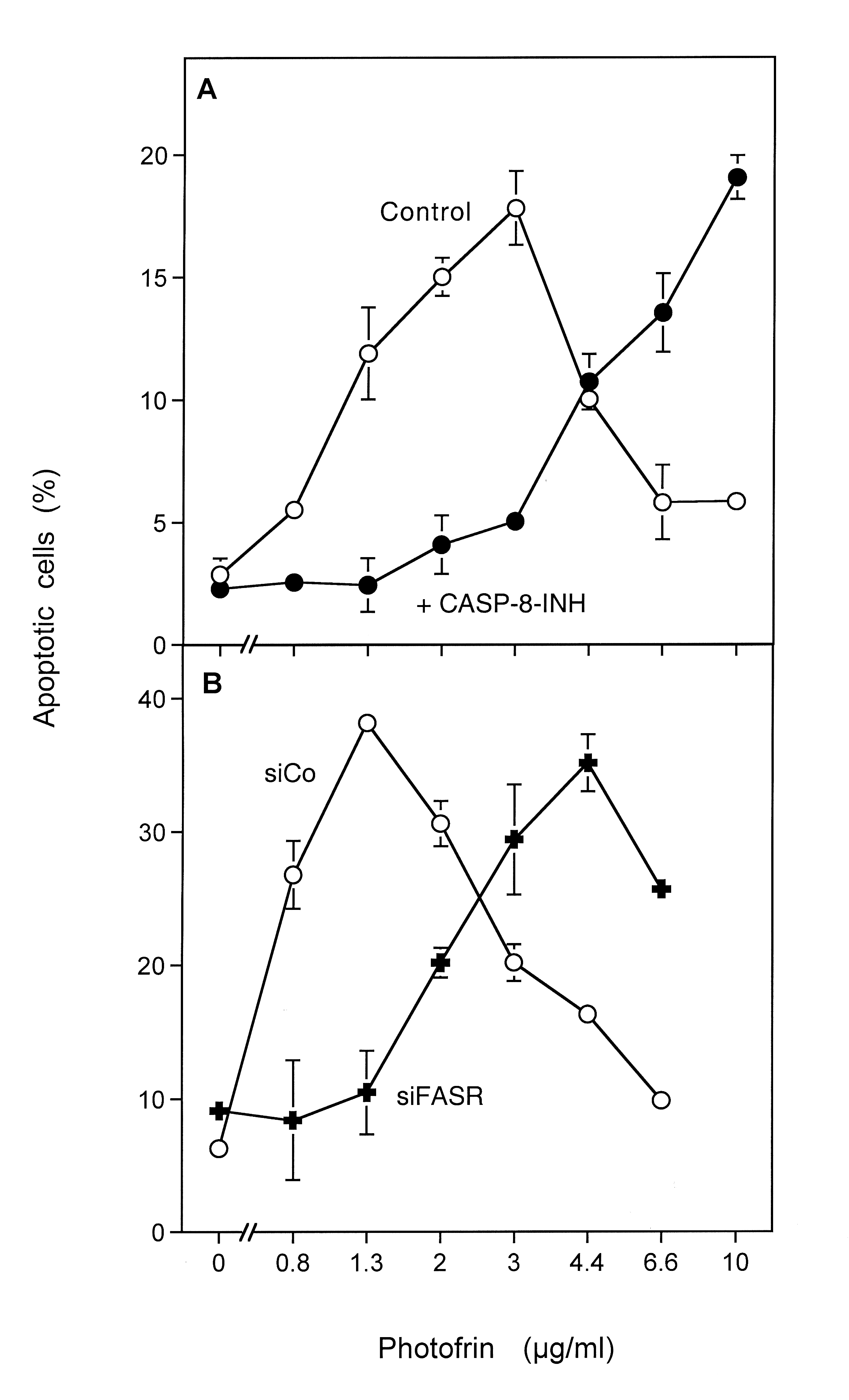


Supplementary Figure 3

**The FAS receptor and caspase-8 are required for optimal reactivation of intercellular ROS-mediated apoptosis signaling triggered by photofrin-derived singlet oxygen**

A. 12 500 MKN-45 cells per assay received 25 µM caspase-8 inhibitor (“+ CASP-8-INH”) or no inhibitor (“control). Photofrin was added at the indicated concentrations, illuminated for 30 min and then the percentages of apoptotic cells were determined after additional 2.5 h.

B. MKN-45 cells were transfected with 24 nM control siRNA (“siCo”) or siRNA directed against the human FAS receptor (“siFASR”) and incubated at 37 °C for 24 h. Cells were washed, resuspended in fresh medium at a density of 12 500 cells/100 µl. The assays received the indicated concentrations of photofrin, were illuminated for 30 min and then the percentages of apoptotic cells were determined after 2.5 h.

Statistical analysis: Apoptosis induction by photofrin and its inhibition by knockdown of the FAS receptor and caspase-8 inhibitor were highly significant (p<0.001).

curve. SiRNA-mediated knockdown of FAS receptor activity caused the same pattern of inhibition of photofrin-mediated apoptosis induction as inhibition of caspase-8, pointing to a central and dominant role of the FAS receptor for the caspase-8-dependent effect during apoptosis induction mediated by low concentrations of photofrin. Prevention of the supraoptimal decline of the optimum curve through caspase-8 inhibitor and knockdown of FAS receptor activity indicates that FAS receptor / caspase-8-dependent processes are involved in excess inactivation of catalase at the supraoptimal site of the induction curve. Excess inactivation of catalase leads to a consumption reaction between high concentrations of H_2_O_2_ and HOCl and thus interferes with apoptosis-inducing signaling (please find details in Bauer, this issue).

As the TRAIL receptor also activates caspase-8, this receptor might contribute to the enhancement of NOX1 activity in the presence of singlet oxygen as well. So far, however, activation by singlet oxygen in a ligand-independent mode has only been described for the FAS receptor (Zhuang et al. 2001). Moreover, the experiment described in Supplementary Figure 3 indicates that FAS receptor activation is sufficient to explain caspase-8-dependent NOX stimulation and its effect on singlet oxygen generation. In addition, unpublished results from our group show that MKN-45 cells express low levels of FAS receptor, but high concentrations of TRAIL receptor. In these cells, apoptosis is induced by TRAIL ligand without the requirement for intercellular ROS signaling, whereas FAS receptor-dependent apoptosis induction depends completely on the reactivation of intercellular ROS signaling. If the TRAIL ligand would be stimulated by singlet oxygen, the experiment presented in Figure 2 D-F (main text of the manuscript) should not show a complete dependence of singlet oxygen-mediated apoptosis induction on the activation of intercellular ROS signaling.

**III. The complexity of singlet oxygen generation in the presence of H_2_O_2_ and peroxynitrite.**

Di Mascio et al. ,1994, have shown that the interaction between H_2_O_2_ and peroxynitrite caused the generation of singlet oxygen. They explained their findings by equation # 1:

. H_2_O_2_ + ONOO^–^ **→** ^1^O_2_ + H_2_O + NO_2_^–^ (equation # 1).

Alvarez et al. ,1995, confirmed that the reaction between H_2_O_2_ + ONOO^–^leads to the formation of oxygen, but did not differentiate between singlet and triplet state oxygen. However, their measurements indicated that two molecules of peroxynitrite were required for the generation of one molecule of oxygen. This is in contradiction to the process described by equation # 1, where one molecule of peroxynitrite would yield one molecule of singlet oxygen. To resolve this discrepancy, the chemistry of peroxynitrite has to be considered in more detail.

Two major alternative reaction pathways are conceivable for peroxynitrite:

A) ONOO**^−^** + H **^+^** → ONOOH (equation # 2)

ONOOH → **.**NO_2_ + **.**OH (equation # 3)

(Beckmann et al., 1990; Goldstein et al., 1999)

B) ONOO**^−^** + CO_2_ → ONOOCOO**^−^** (equation # 4)

ONOOCOO**^−^** → **.**NO_2_ + CO_3_**^. −^** (equation # 5).

(Denicola et al., 1996; Goldstein and Czapski, 1998; Squadrito and

Prior, 1998; Augusto et al., 2002; Espay et al., 2002;)

Hydroxyl radicals (**.**OH) as well as carbonate radicals (CO_3_**^. −^**) can react with H_2_O_2_:

**.**OH + H_2_O_2_ → HO_2_**.** + H_2_O (equation # 6)

(Christensen et al., 1982; Buxton et al., 1988)

CO_3_**^. −^** + H_2_O_2_ → HO_2_**.** + HCO_3_ **−** (equation #7)

(Goldstein and Czapski, 1998)

and both reactions yield perhydroxyl radicals (HO_2_**.**) which can generate singlet oxygen either through the reaction

2 HO_2_**.** → H_2_O_2_ + ^1^O_2_ (equation #8, reaction rate 8.6 x 10^5^ M^-1^ s ^-1^)

or the faster reaction

HO_2_**.** + O_2_**^. −^** + H^+^ → H_2_O_2_ + ^1^O_2_ (equation #9, reaction rate 9.7 x 10^7^ M^-1^ s ^-1^)

(Fridovich 1975; Aurand et al., 1977; Badway and Karnovsky, 1980;

Tarr and Valenzeno, 2003).

Therefore, both reaction pathways of peroxynitrite (equations # 2/3 and equations # 4/5) in combination with the reactions between either hydroxyl radicals or carbonate radicals with H_2_O_2_ might finally lead to the generation of singlet oxygen. Both reaction schemes would be in line with the inhibitor profile shown in Figure 5 (main manuscript) that indicates that peroxynitrite and H_2_O_2_ are required for singlet oxygen generation, and that peroxynitrite and H_2_O_2_ are derived from tumor cell-derived superoxide anions and NO.

For further clarification of this issue, MKN-45 cells were treated with photofrin in the absence or presence of the NOX1 inhibitor AEBSF, the hydroxyl radical scavenger mannitol or the carbonate radical scavenger tryptophan. They were then challenged with exogenous peroxynitrite to quantify the inactivation of membrane-associated catalase. As shown in Supplementary Figure 4 A, photofrin sensitized the tumor cells for the apoptosis-inducing effect of peroxynitrite, indicating the inactivation of membrane-associated catalase. In line with our previous findings, the presence of AEBSF prevented catalase inactivation, pointing to the necessity of superoxide anion-driven processes that amplify the generation of cell-derived singlet oxygen after the application of exogenous singlet oxygen, as outlined in Figure 8 (main manuscript). These processes were abrogated at lower cell density (Supplementary Figure 4 A) and therefore seem to be due to intercellular signaling. The presence

*Supplementary Figure 4*


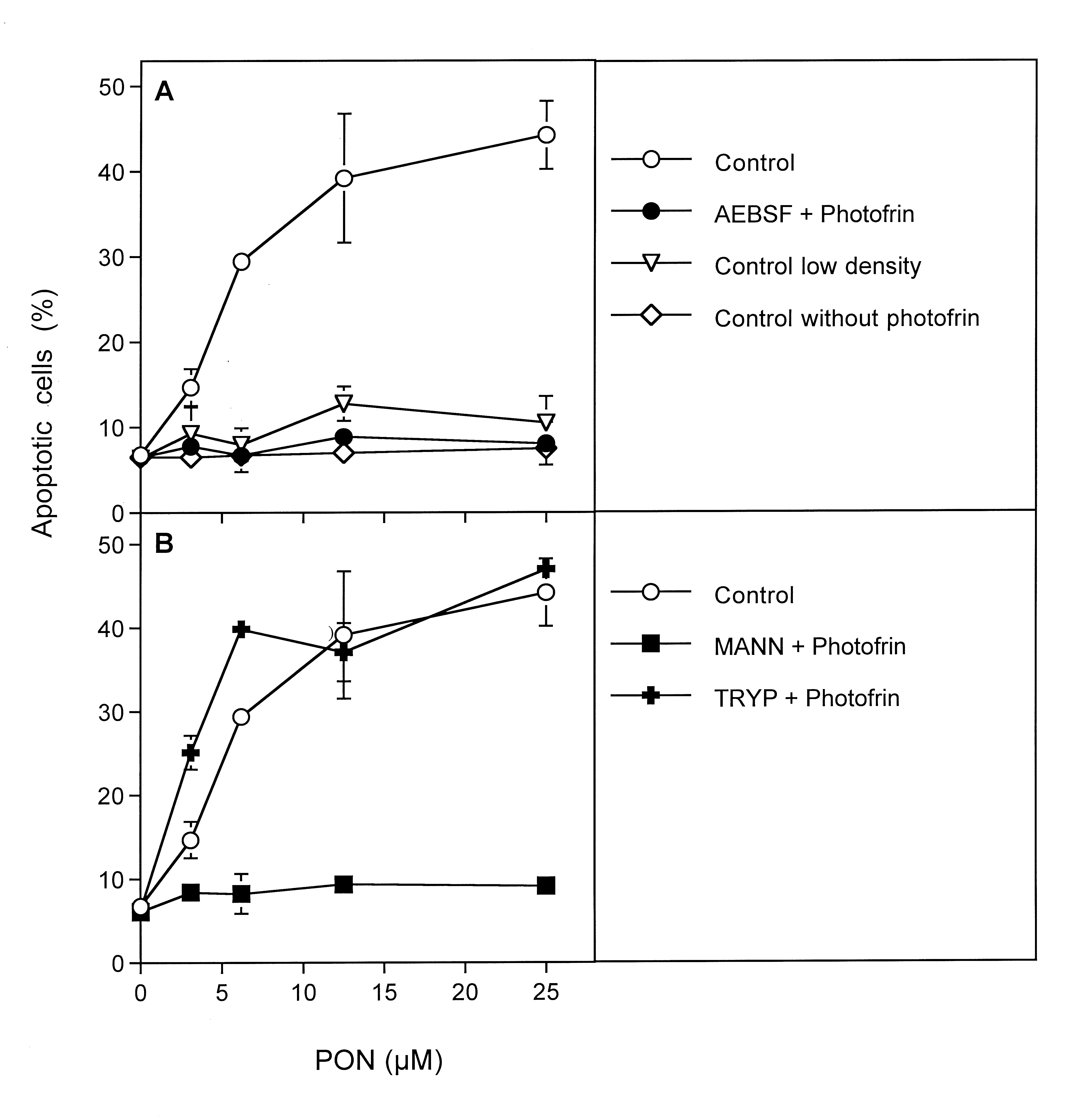


Supplementary Figure 4: **Hydroxyl radicals are involved in the formation of singlet oxygen through the interaction between H_2_O_2_ and peroxynitrite.**

125 000 MKN-45 cells/ml received no inhibitor (control) or 100 µM of the NOX inhibitor AEBSF, 20 mM of the hydroxyl radical scavenger mannitol (MANN) or 20 mM of the carbonate radical scavenger tryptophan (TRYP) before 10 µg/ml photofrin were added and the assays were illuminated with visible light for 20 min. Additional controls contained 40 000 cells plus photofrin (control low density). After illumination, the assays received 100 µM AEBSF and were washed three times, using medium containing 100 µM AEBSF. The cells were resuspended in medium plus AEBSF at a density of 10 000 cells / 100 µl and received the indicated concentrations of peroxynitrite. Additional controls had not been treated with photofrin, but received peroxynitrite. After 1.5 h, the percentages of apoptotic cells were determined in duplicate assays.

Statistical analysis: Apoptosis induction by peroxynitrite and the inhibition by mannitol, as well as the inhibitory effect of low cell density were highly significant (p<0.001).

of the carbonate radical scavenger tryptophan did not prevent catalase inactivation after singlet oxygen treatment, but the hydroxyl radical scavenger mannitol completely inhibited catalase inactivation. This finding shows that the amplification of singlet oxygen generation based on H_2_O_2_ and peroxynitrite seems to follow equations #2, #3, #6, #8 and/or #9 rather than equations #4, #5, #7, #8 and/or #9.

Due to the abundance of CO_2_ and its derivatives in the medium and in biological systems, one might expect the reaction described by equation # 4 should be heavily competing with the reaction described by equation # 2. However, we have shown that proton pumps facilitate the reaction described by equation #2, especially in close vicinity to the membrane of tumor cells and thus contribute significantly to the apoptosis-inducing effect of peroxynitrite that is mediated by hydroxyl radicals (Bauer, this issue). If our conclusions were correct, the proton pump inhibitor omeprazole and the hydroxyl radical scavenger mannitol should not only prevent

peroxynitrite-dependent apoptosis, but also singlet oxygen generation through peroxynitrite/H_2_O_2_ interaction. Supplementary Figure 5 shows that our conclusions were correct. Addition of mannitol (Supplementary Figure 5 A) or omeprazole (Supplementary Figure 5 B) prior to the addition and illumination of photofrin had a strong inhibitory effect on catalase inactivation (determined by a peroxynitrite challenge). These inhibitory effects were nearly completely abrogated when the

inhibitors had been added 20 min after photofrin, immediately before the assays were washed, followed by the peroxynitrite challenge. This control ensures that the

*Supplementary Figure 5*


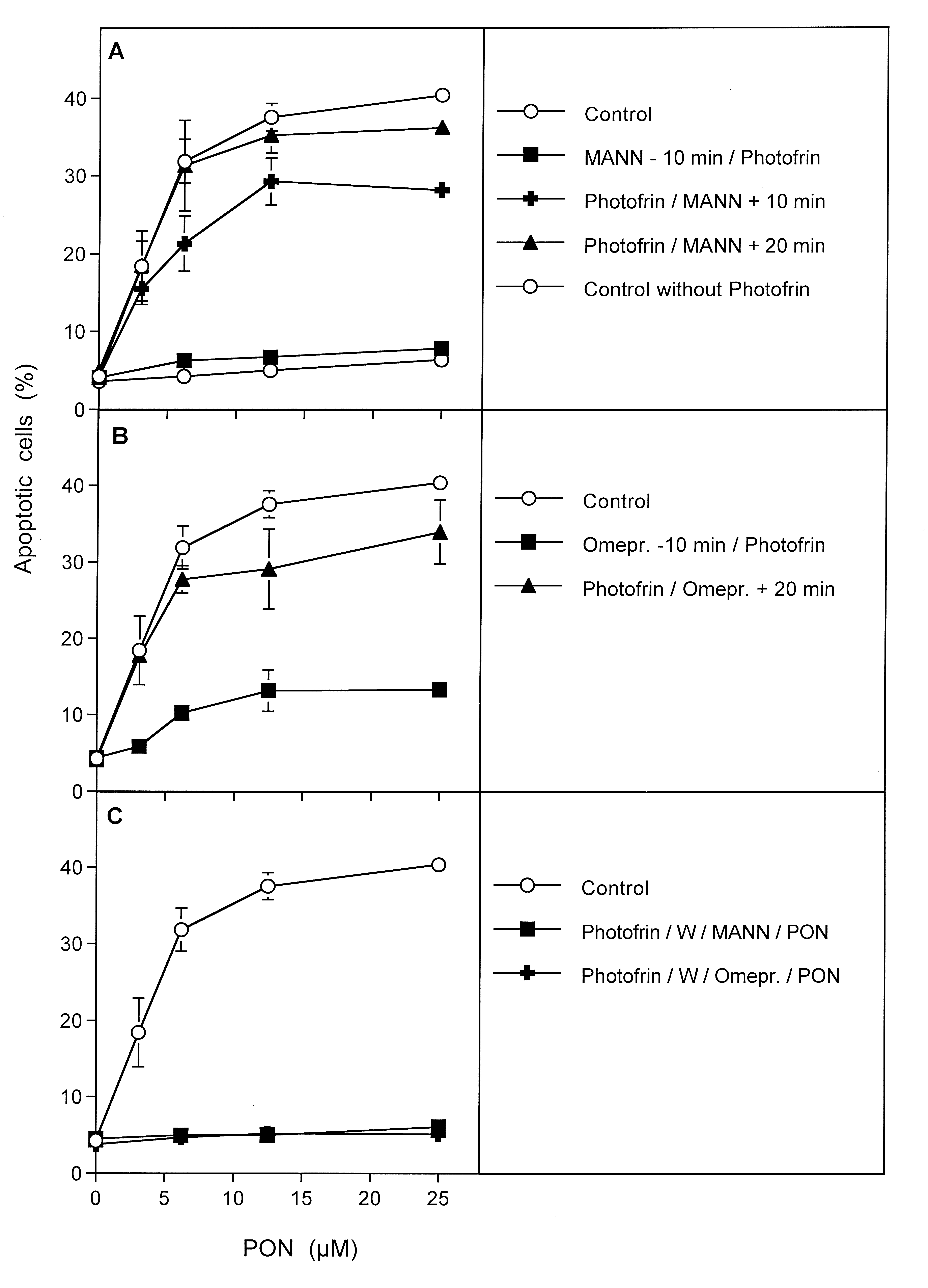


Supplementary Figure 5: **The role of protons and hydroxyl radicals for the formation of singlet oxygen through the interaction between H_2_O_2_ and peroxynitrite.**

A, B: 125 000 MKN-45 cells/ml received either no inhibitor (control) or 20 mM of the hydroxyl radical scavenger mannitol or 9 µM of the proton pump inhibitor omeprazole 10 min before 10 µg/ml photofrin were added and illuminated for 20 min (“Mann – 10 min / Photofrin”; “Omepr. -10 min / Photofrin”). Additional assays received mannitol or omeprazole 10 min or 20 min after the addition and illumination of photofrin (“Photofrin/MANN + 20 min”; Photofrin /OMepr. + 20 min”). After illumination, the assays received 100 µM AEBSF and were washed three times, using medium containing 100 µM AEBSF. The cells were resuspended in medium plus AEBSF at a density of 10 000 cells / 100 µl and received the indicated concentrations of peroxynitrite. Additional controls had not been treated with photofrin, but received peroxynitrite. After 1.5 h, the percentages of apoptotic cells were determined in duplicate assays.

C. 125 000 MKN-45 cells/ml received 10 µg/ml photofrin and were illuminated for 20 min (“Mann – 10 min / Photofrin”; “Omepr. -10 min / Photofrin. After illumination, the assays received 100 µM AEBSF and were washed three times, using medium containing 100 µM AEBSF. The cells were resuspended in medium plus AEBSF at a density of 10 000 cells / 100 µl and received either 20 mM mannitol (“Photofrin / W / MANN / PON”) or 9 µM omeprazole (“Photofrin / W / Omepr / PON”) before the indicated concentrations of peroxynitrite were added. After 1.5 h, the percentages of apoptotic cells were determined in duplicate assays.

Statistical analysis: Apoptosis induction in photofrin-treated cells by peroxynitrite, as well as its inhibition by mannitol and omeprazole added prior to photofrin was highly significant (p<0.001), whereas later addition of inhibitors to photofrin-containing assays caused no significant inhibitory effect. Inhibition of peroxynitrite-dependent apoptosis by inhibitors added after photofrin- and before peroxynitrite treatment was highly significant (p<0.001).

washing procedure was effective and that the inhibitory effect of mannitol and omeprazole (being present before and together with photofrin) were not due to residual inhibitor that interfered with the peroxynitrite challenge. Finally, Supplementary Figure 5 C confirms that apoptosis induction by peroxynitrite depends on protonation of peroxynitrite and hydroxyl radical generation through its homolysis, as addition of omeprazole or mannitol after photofrin treatment and the wash step, but before addition of peroxynitrite, completely blocked apoptosis induction.

To rule out an effect of omeprazole on superoxide anion generation that might reduce the concentration of available H_2_O_2_, the effect of omeprazole on superoxide anion generation was tested. As shown in Supplementary Figure 6, omeprazole up to 10 µM had no inhibitory effect on superoxide anion generation. As we had used 9 µM of the compound in our experiments, an effect on superoxide anion and H_2_O_2_ concentration can therefore be ruled out.

Taken together our data indicate that singlet oxygen generation through the interaction between peroxynitrite and H_2_O_2_ most likely is not due to direct interaction between these two molecules, but seems to be mediated by hydroxyl radicals that are derived from peroxynitrous acid and that react with hydrogen peroxide. The resulting perhydroxyl radicals then can generate singlet oxygen either through direct interaction or through interaction with superoxide anions and protons. This sequence is in line with our inhibition data, with the stocheometric findings by Alvarez et al., 1995 and with the observation by Di Mascio et al., 1994, that singlet oxygen is generated when peroxynitrite and H_2_O_2_ are present.

*Supplementary Figure 6*


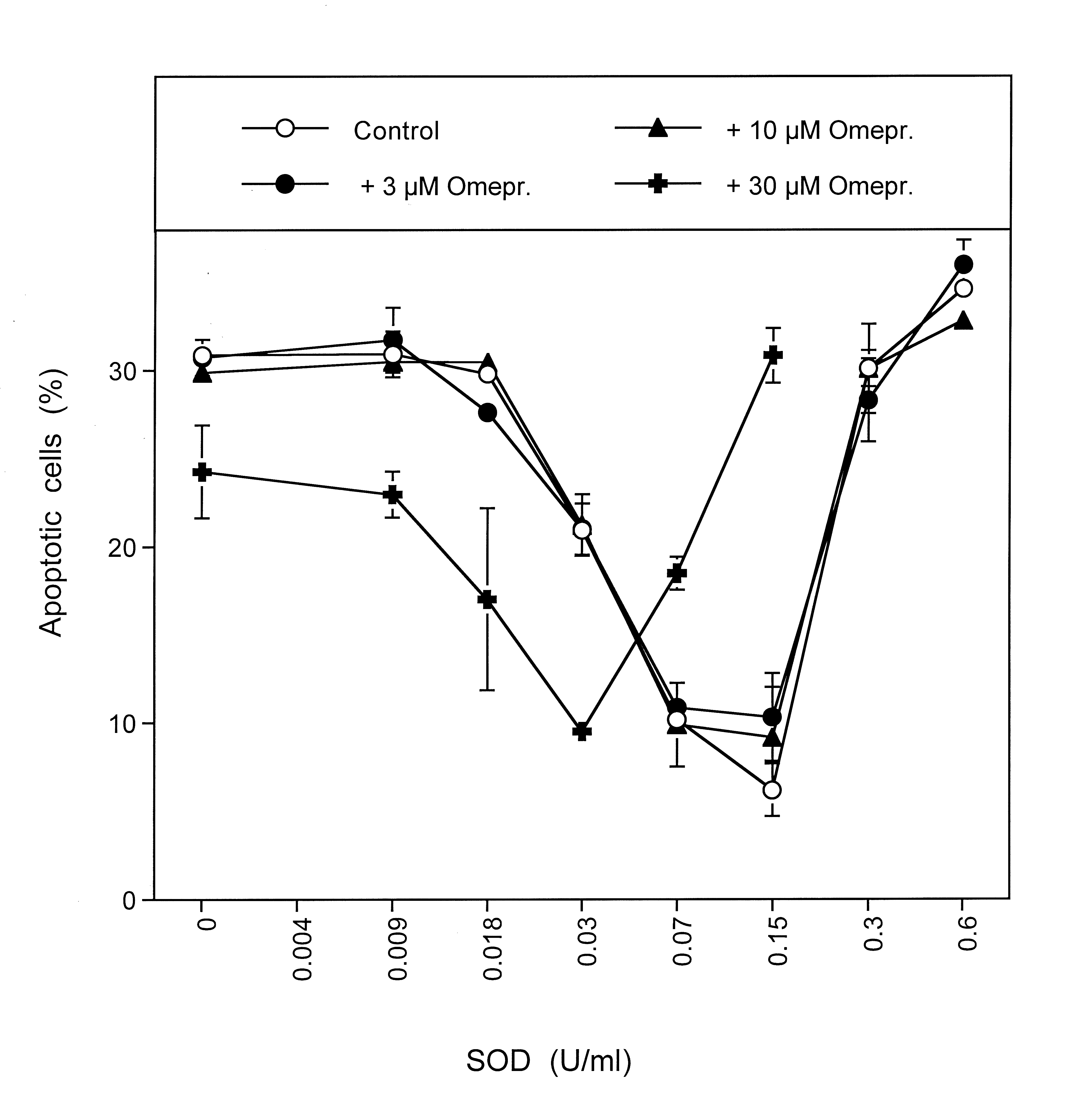


Supplementary Figure 6: **Effect of omeprazole on superoxide anion generation**

12 500 MKN-45 cells / 100 µl contained 125 mM of the catalase inhibitor 3-AT and the indicated concentrations of omeprazole. The assays received the indicated concentrations for Cu/ZnSOD and were incubated at 37 °C for 5 h. The percentages of apoptotic cells were determined after 5 h in duplicate assays. As recently described, the bell-shaped inhibition curve is indicative for the relative concentration of superoxide anions in the system (Temme and Bauer, 2013). The result shows that omeprazole up to a concentration of 10 µM does not affect superoxide anion generation, whereas 30 µM omeprazole cause a reduction of superoxide anion production to 25 %.

Statistical analysis: Omeprazol up to 10 µM did not cause a significant increase in superoxide anion generation, whereas the inhibitory effect of 30 µM omeprazole was highly significant (p<0.001).

**IV. Supplementary Materials and Methods**

*SiRNA-mediated knockdown of the FAS receptor.*

*SiRNAs.* Control siRNA (“siCo”) and siRNA for the knockdown of the human FAS receptor (siFASR”) were obtained from Qiagen, Hilden, Germany.

Sequences of siRNAs used:

1) siCo:

sense: r(UUCUCCGAACGUGUCACGU)dTdT

antisense: ACGUGACACGUUCGGAGAA)dTdT

SiCo was determined by the manufacturer as not affecting the expression of any known gene.

2) siFASR for the knockdown of the human FAS receptor *(Apo-1, CD95)*

Hs_FAS_7_HP Validated siRNA

Cat. Nr. SI02654463

Target Sequence: AAG GAG TAC ACA GAC AAA GCC

*Transfection.* SiRNAs were dissolved in suspension buffer supplied by Qiagen at a concentration of 20 µM. Suspensions were heated at 90 °C for 1 minute, followed by incubation at 37°C for 60 minutes. Aliquots were stored at -20 °C.

Before transfection, 88 µl of medium without serum and without antibiotics were mixed with 12 µl Hyperfect solution (Qiagen, Hilden, Germany) and 1.2 µl of siRNA. The mixture was treated by a Vortex mixer for a few seconds and then allowed to sit for 10 minutes. It was then gently and slowly added to 300 000 MKN-45 cells in 1ml RPMI 1640 medium containing 10 % FBS and antibiotics. The cells were incubated at 37 °C, 5 % CO_2_ for 24 hours. The cells were collected, centrifuged and resuspended in fresh medium at the desired concentration.

*Transfection efficiency and knockdown of the FAS receptor.* Control experiments showed that the transfection efficiency was much greater than 90% when Hyperfect transfection reagent (Qiagen) and the protocol summarized above were used (data not shown). Validation of the siRNA directed against the FAS receptor by the supplier using RT PCR showed that it reduced the expression of FAS receptor mRNA more than 80 %. Functional analysis of FAS receptor knockdown showed that treatment with 25 nM siRNA for 24 h caused complete loss of response to apoptosis-inducing antibodies directed against the FAS receptor or to FAS ligand, whereas control siRNA did not affect apoptosis induction (Bauer, submitted, this issue) .

**V. Supplementary References**

Alvarez B, Denicola A and Radi R: Reaction between peroxynitrite and hydrogen peroxide: Formation of oxygen and slowing of peroxynitrite decomposition. Chem. Res. Toxicol. 8: 859-864, 1995.

Augusto O, Bonini MG, Amanso AM, Linares E, Santos CX and De Menzes SL: Nitrogen dioxide and carbonate radical anion: two emerging radicals in biology. Free Rad Biol Med 32: 841-859, 2002.

Aurand LW, Boone NN and Giddings GG: Superoxide and singlet oxygen in milk lipid peroxidation. J Dairy Science 60: 363-369, 1977

Badway JA and Karnovsky ML: Active oxygen species and the functions of phagocytic leukocytes. Ann Rev Biochem 49: 695-726, 1980.

Beckman JS, Beckman TW, Chen J, Marshall PA and Freeman BA. Apparent hydroxyl radical production by peroxynitrite: implications for endothelial injury form nitric oxide and superoxide. Proc. Natl. Acad. Sci. U.S.A. 87: 1620-1624, 1990.

Bodmer J-L, Holler N, Reynard S, Vinciguerra P, Schneider P, Juo P, Blenis J and Tschopp J. Trail receptor-2 signals apoptosis through FADD and caspase-8. Nature Cell Biol 2: 241-243, 2000.

Buxton GV, Greenstock CL, Helman WP and Ross AB. Critical reviews of rate constants for reactions of hydrated electrons, hydrogen atoms and hydroxyl radicals (**.**OH/**.**O^−^) in aqueous solution. J Phys Chem Reference Data 17: 513-886, 1988

Christensen H, Sehested K and Corfitzen H: Reactions of hydroxyl radicals with hydrogen peroxide at ambient and elevated temperature. J Phys Chem 86: 1588-1590, 1982.

De Milito A and Fais S: Tumor acidity, chemoresistance and proton pump inhibitors. Future oncology 1: 779-786, 2005.

Denicola A, Freeman BA, Trujillo M and Radi R. Peroxynitrite reaction with carbon dioxide/bicarbonate: kinetics and influence on peroxynitrite-mediated reactions. Arch Biochem Biophys 333: 49-58, 1996.

Di Mascio P, Bechara EJH, Medeiros MHG, Briviba K, and Sies H. Singlet molecular oxygen production in the reaction of peroxynitrite with hydrogen peroxide. *FEBS Letters* 355: 287-289, 1994

Espey MG, Miranda KM, Thomas DD, Xavier SA, Citrin D, Vitek MP and Wink DA: A chemical perspective on the interplay between NO, reactive oxygen species, and reactive nitrogen species. Ann. N.Y. Acad. Sci. 962: 195-206, 2002.

Fridovich I: Superoxide dismutases. Ann Rev Biochem 44: 147-159, 1975.

Goldstein S and Czapski G: Formation of peroxynitrate from the reaction of peroxynitrite with CO_2_: Evidence for carbonate radical production. J Am Chem Soc 120: 3458-3463, 1998.

Goldstein S, Meyerstein D, van Eldik R and Czapski G. Peroxynitrous acid decomposes via homolysis: evidence from high-pressure pulse radiolysis. J. Phys. Chem. A 103: 6587-6590, 1999.

Squadrito GL and Pryor WA. Oxidative chemistry of nitric oxide: the roles of superoxide, peroxynitrite, and carbon dioxide. Free Rad Biol & Med 25: 392-403, 1998.

Tarr M and Valenzeno DP: Singlet oxygen: the relevance of extracellular production mechanisms to oxidative stress in vivo. Photochem Photobiol Sci 2: 355-361, 2003

Temme J and Bauer G: Low-dose gamma irradiation enhances superoxide anion production by nonirradiated cells through TGF-β1-dependent bystander signaling. Rad. Res. 179: 422-432, 2013

Zhuang S, Demir JT and Kochevar IE: Protein kinase C inhibits singlet oxygen-induced apoptosis by decreasing caspase-8 activation. Oncogene *20*: 6764-6776, 2001.
